# Hybrid Fluorescent Poly(silsesquioxanes) with Amide- and Triazole-Containing Side Groups for Light Harvesting and Cation Sensing

**DOI:** 10.3390/ma13204491

**Published:** 2020-10-10

**Authors:** Maria Nowacka, Tomasz Makowski, Anna Kowalewska

**Affiliations:** Centre of Molecular and Macromolecular Studies, Polish Academy of Sciences, Sienkiewicza 112, 90-363 Łódź, Poland; tomekmak@cbmm.lodz.pl (T.M.); anko@cbmm.lodz.pl (A.K.)

**Keywords:** poly(silsesquioxanes), fluorescence, optical activity, pyrene, fluorescence energy transfer, cation sensing

## Abstract

Hybrid polymers containing pyrene (Py) units bound to linear poly(silsesquioxane) (LPSQ) chains through flexible linkers containing heteroatoms (S, N, O) (LPSQ-triazole-Py and LPSQ-amide-Py) exhibit intense fluorescence emission, both in very diluted solutions (c = 10^−8^ mol/L) and in the solid state. The materials are thermally stable and exhibit good thin film forming abilities. Their optical and physicochemical properties were found to be strongly dependent on the structure of the side chains. Comparative studies with octahedral silsesquioxane (POSS) analogues (POSS-triazole-Py and POSS-amide-Py) emphasized the role of the specific double-strand architecture of the LPSQ backbone and distribution of side Py groups for their photo-luminescent properties. The new hybrid materials were tested as fluorescence energy donors to red-emitting dyes (Nile Red and Coumarine 6). All the silsesquioxanes studied were found to be able to transfer FL emission energy to Coumarin 6, irrespectively of their spatial structure. However, due to the differences in the wavelength range of FL emission, only LPSQ-triazole-Py were able to act as energy donors to Nile Red. The Py-grafted LPSQ may be also applied for development of soluble and highly emissive chemosensors. Their fluorescent nature was explored for the detection of Cu(II), Fe(III), Co(II), Ag(I), Hg(II), Mg(II), Ca(II), Pb(II) and Zn(II). The morphology of the side chains and hydrogen-bonding interactions influenced the sensing capacity of all the studied materials.

## 1. Introduction

With fast growing awareness of environmental issues, an increasing level of pollution in the natural environment and the search for new reproducible energy sources, the demand for technologies has been shifted to novel intelligent materials. Organic light-emitting diodes [1], organic photovoltaics [2], field effect transistors [3] or biosensors are the focus of these studies and potential applications. Derivatives of pyrene (Py) are organic π-conjugated molecules that exhibit strong absorbance, high quantum yield and good photochemical stability [4,5]. Those optoelectronic properties may be of advantage in light harvesting systems based on nonradiative excited state energy transfer (EnT) from antenna chromophores to acceptor molecules in the ground state [6,7]. The EnT in artificial light-harvesting systems occurs through Förster resonance energy transfer (FRET) [8].

Green light emitting Py excimers are formed when geometrical overlap of aromatic rings is provided. Highly organized systems, such as crystals of Py-containing molecules emit almost exclusively excimer fluorescence in the solid state [9]. Yet, preparation of thin fluorescent films that would exhibit high Py excimer emission may be a problem. Luminescence decrease or quenching is quite often observed at high concentrations (in solution and in solid amorphous state) due to formation of nanoaggregates. Such self-quenching phenomena may challenge functions of OLED’s or artificial light-harvesting antenna [10].

Fluorescence spectroscopy is also widely used for environmental and biological studies due to the low detection limit, selectivity, rapid response and applicability for bioimaging [11,12,13]. Py derivatives being highly effective fluorophores are frequently used as fluorescent probes (e.g., for detection of changes in temperature [14], pressure [15], pH [16], concentration of gases [17], organic molecules [18], inorganic anions [19] and metal cations [20]). The drawback of the use of low molecular weight pyrene derivatives is that efficient interactions between chromophores occur at high concentrations. Under such conditions, Py fluorescence is quite often quenched.

Therefore, of high interest are novel molecules or macromolecules that allow for efficient use of Py fluorophores in dilute solutions and suppress π-stacking-induced molecular aggregation of pyrene in solid state and concentrated solutions. One of the possible resolutions is preparation of Py labeled polymer materials. Various types of optically active polymers have been prepared to date—e.g., Py functionalized polyacetylenes [21], poly(N-vinyl-2-pyrrolidone) [22], copolymers prepared though radical polymerization of n-butyl methacrylate and Py coupled methacrylates [23], poly(1-pyrenylmethoxycarbonylmethylene) [24], poly(methoxy diethylene glycol methacrylate) [25] and polystyrene [26]. Additionally, Py containing silicon-based materials have been prepared recently. Octavinylsilsesquioxane functionalized with Py exhibited good optical properties in solution and solid state and was successfully tested for application in electroluminescent devices [27,28]. However, properties of polyhedral silsesquioxanes (poor film forming properties, crystal structure, low molecular weight) exclude them from some applications, such as production of large area displays. Grafting pyrene onto poly(siloxanes) [29] resulted in better properties and expanded the area of their possible applications. Due to the unique architecture and properties, even better results can be achieved with linear poly(silsesquioxanes), but not much research has been dedicated to their preparation to date. We have recently shown that optically active linear poly(2-(pyren-1-yl)vinyl)silsesquioxanes (LPSQ-Py) bearing Py moieties in their side chains are highly emissive, even in very dilute solutions [30]. They exhibited almost exclusively intramolecular excimer emission and scarce fluorescence quenching. The effect was ascribed to the fixed position of Py groups linked with rigid spacers to the double-strand silsesquioxane backbone.

It can be thus assumed that the flexibility and interactions between the side chains linking Py and LPSQ backbone may influence interactions between chromophores. In this study, we present a new type of linear polysilsesquioxanes, LPSQ-R-Py, bearing nucleophilic oxygen, nitrogen or sulphur atoms embedded in the spacers (-R-) between the polymer backbone and Py moieties. We thoroughly studied the influence of the side chains structure with regard to the optical properties of LPSQ (characteristic position of FL bands and intensity of emission) both in dilute solutions and in the solid state. In order to determine the role of characteristic architecture of LPSQ, we also studied properties of their octahedral core silsesquioxane analogues (polyhedral oligomeric silsesquioxanes, POSS).

The improved efficiency of interactions between side chromophores in LPSQ-R-Py allowed for efficient EnT to organic dyes [9-diethylamino-5-benzo[α]phenoxazinone (Nile Red) and 2H-chromen-2-one (Coumarin 6)]. The effect of different paramagnetic [Cu(II), Fe(III), Co(II)] and diamagnetic [Ag(I), Hg(II), Mg(II), Ca(II), Pb(II), Zn(II)] metal cations on optical properties of the LPSQ-R-Py was also examined. Materials with triazole linkers in side groups exhibited high sensitivity toward Cu(II) and Ag(I) ions, and only slightly poorer sensitivity for Hg(II) and Fe(III). LPSQ-amide-Py detected Co(II), Fe(III) and Cu(II), yet the effect was not large. POSS-amide-Py was selective toward Co(II).

## 2. Materials and Methods

### 2.1. Materials

Linear poly(vinylsilsesquioxanes) of regular structure and triethylsilyl end groups (LPSQ-Vi; Mn = 1000 g/mol, PDI = 1.2) were prepared according to the published procedure [31]. 1,3,5,7,9,11,13,15-Octavinylpentacyclo-[9.5.1.13,9.15,15.17,13]octasiloxane (POSS-Vi) was purchased from Hybrid Plastics (Hattiesburg, MS, USA) and used as received. Linear LPSQ-TG were prepared following a literature procedure [32]. POSS-TG was prepared in an analogous way. LPSQ-amide-Py and POSS-amide-Py were prepared through condensation of side -COOH groups of LPSQ-TG or POSS-TG with 1-aminopyrene. Silsesquioxanes bearing 3-chloropropyl side groups were prepared following the literature procedure (LPSQ-Cl [33] and POSS-Cl [34]). LPSQ-N_3_ and POSS-N_3_ were obtained via azidation of LPSQ-Cl and POSS-Cl, respectively. LPSQ-triazole-Py and POSS-triazole-Py were obtained via the copper(I)-catalyzed Huisgen cycloaddition of 1-ethynylpyrene to LPSQ-N_3_ or POSS-N_3_. (3-Azidopropyl)trimethoxysilane was prepared according to the modified literature procedure [35].

The detailed synthetic procedure and products characterization can be found in Supplementary Information.

Commercially available reagents (1-ethynylpyrene (96%, ABCR, Karlsruhe, Germany), 1-aminopyrene (95%, ABCR, Karlsruhe, Germany), N,N,N′,N″,N″-pentamethyldiethylenetriamine (PMDETA, 99% Aldrich, Poznań, Poland), copper bromide (98%, Alfa Aesar, Lancashire, UK), silica gel applied as a reagent and column chromatography solid phase (high purity grade, pore size 60Å, 70–230 mesh; Aldrich, Poznań, Poland), potassium iodide (99%, Aldrich, Poznań, Poland), sodium azide (99% extra, Acros Organics, Geel, Belgium), thioglycolic acid (>99%, Aldrich, Poznań, Poland), MgSO_4_ (pure, Chempur, Piekary Śląskie, Poland)), 2,2-dimethoxy-2-phenylacetophenone (DMPA; 99%, Acros Organics, Geel, Belgium)), p-phenylenediamine (pPDA, >99%, Aldrich, Poznań, Poland), triethylamine (>98%, Fluka, Gillingham, UK), HCl (36–38%, Stanlab Sp. J., Lublin, Poland), 3-chloropropyltrichlorosilane (for synthesis; Aldrich, Poznań, Poland) and fluorescent dyes ((9-diethylamino-5-benzo[α]phenoxazinone, Nile Red, extra pure; Roth, Worcester, UK) and 3-(2-Benzothiazolyl)-7-(diethylamino)coumarin (Coumarin 6; 98%, Aldrich, Poznań, Poland)) were used as received.

Solvents used for the synthesis (tetrahydrofuran (THF) (Chempur, pure p.a.), dichloromethane (Chempur, pure p.a.), methanol (p.a., Chempur, Piekary Śląskie, Poland), toluene (p.a., Chempur, Piekary Śląskie, Poland)) and N,N-dimethylformamide (DMF; p.a., Chempur, Piekary Śląskie, Poland) were purified according to literature procedures [36]. Solvents used for spectroscopic studies (FL and absorption analysis): THF (spectroscopic grade; Alfa Aesar, Lancashire, UK) and CHCl_3_ (spectroscopic grade; Uvasol Merck, Darmstadt, Germany) were used as received. 

### 2.2. Instrumentation

#### 2.2.1. Nuclear Magnetic Resonance (NMR)

Liquid state ^1^H NMR, ^13^C NMR and ^29^Si NMR spectra were recorded in CDCl_3_, CD_3_OD or THF-d_6_ on a Bruker DRX-500 MHz spectrometer. Solid-state ^13^C NMR and ^29^Si NMR spectra were recorded on BRUKER Avance III 400 spectrometer (Bruker, Billerica, MA, USA) with high power decoupling (HP Dec) of direct polarization.

#### 2.2.2. Thermal Analysis

Thermogravimetric analysis (TGA) of the prepared materials was performed in nitrogen atmosphere (heating rate 10 °C/min, resolution 3, sensitivity 3) using a Hi-Res TGA 2950 Thermogravimetric Analyzer (TA Instruments, New Castle, DE, USA) with a platinum heating pan.

The samples were also studied using differential scanning calorimetry (DSC 2920 Modulated TA Instruments). Thermograms were taken for samples (sealed in aluminum pans) heated under nitrogen at the rate of 10 °C/min from room temperature to 200 (LPSQ/POSS-Cl), 180 (LPSQ/POSS-N_3_), 150 (LPSQ/POSS-triazole-Py) or 100 °C (LPSQ/POSS-amide-Py, LPSQ/POSS-Vi). The samples were then cooled at 10 °C/min to −50 °C and heated again. The heating/cooling cycles were repeated trice.

#### 2.2.3. Fourier-Transform Infrared Spectroscopy (FTIR)

Fourier transform infrared spectra were recorded with a Nicolet 380 FTIR spectrometer (Thermo Scientific, Waltham, MA, USA) for thin films of all products cast on crystal KBr windows. The technique of attenuated total reflectance (ATR) was applied for IR measurements, using a deuterated triglycine sulfate (DTGS) detector with a diamond ATR accessory Golden Gate (Nicolet 6700 spectrometer; Thermo Fisher Scientific, Waltham, MA, USA). The spectra were obtained by adding 64 scans at a resolution of 2 cm^−1^.

#### 2.2.4. Wide Angle X-Ray Scattering (WAXS)

Wide-angle X-ray scattering (WAXS) measurements were performed using a CuKα radiation source (Philips, Guildford, UK), operating at 30 kV and 50 mA. The diffraction patterns were recorded in the 2Θ scattering range from 1 to 37°. The typical time of acquisition was 7 sec. Samples were irradiated at the incident angle (αi) of 0.05°.

Measurements were carried out for thin layers prepared by the drop casting method from solutions in chloroform (LPSQ/POSS-triazole-Py) or DMF (LPSQ/POSS-amide-Py), c_Py_ = 10^−3^ mol/L.

#### 2.2.5. Atomic Force Microscopy (AFM)

Morphology of thin films of LPSQ/POSS-triazole-Py and LPSQ/POSS-amide-Py was investigated with a Flex Axiom atomic force microscope with C3000 controller (Nanosurf AG, Liestal, Switzerland). The measurements were performed in air, using a noncontact mode. All of the images were prepared with a resolution of 512 × 512 data points.

Measurements were carried out for thin layers prepared by drop casting from solutions in chloroform (LSPQ/POSS-triazole-Py) and DMF (LPSQ/POSS-amide-Py), c_Py_ = 10^−3^ mol/L.

#### 2.2.6. Molecular Modelling

Models of molecules of LPSQ/POSS-triazole-Py and LPSQ/POSS-amide-Py were constructed on the HyperChem platform [37]. The atomic point charges were calculated using the semi-empirical AM1 method and geometry was optimized by the molecular mechanics method (using MM+ force field parameter set with Polak–Ribiere energy minimization algorithms).

#### 2.2.7. Reflected Light Microscopy

Thin films cast on silicon plates from solutions in DMF (c_Py_ = 10^−3^ mol/L) were analyzed by reflected light microscopy with a Delta Optical light microscope (Delta Optical, Warszawa, Poland) equipped with a digital camera.

#### 2.2.8. Ultraviolet–Visible Spectroscopy (UV)

UV–VIS measurements were performed using a Specord S600 spectrophotometer, associated with Win Aspect software version 2.3 (Analytik Jena AG, Jena, Germany).

Solutions of LPSQ/POSS-triazole-Py and LPSQ/POSS-amide-Py at different concentrations (c_Py_ = 10^−8^ − 10^−3^ mol/L) were prepared in THF, CHCl_3_ and DMF and placed in 10 mm path length quartz cuvettes. Measurements in solid state were carried out for thin films spin coated on quartz plates from solutions of LPSQ/POSS-triazole-Py and LPSQ/POSS-amide-Py in chloroform (c_Py_ = 10^−6^ mol/L).

#### 2.2.9. Fluorescence Spectroscopy (FL)

Fluorescence spectra were recorded at room temperature using selected excitation wavelengths with a Horiba Jobin Yvon, Fluorolog-3 spectrofluorometer (Horiba Jobin Yvon, Kioto, Japan). The slit size used for excitation and emission measurements was 2 nm. Solutions of LPSQ/POSS-triazole-Py and LPSQ/POSS-amide-Py at different concentration (c_Py_ = 10^−8^ − 10^−3^ mol/L) were prepared in THF, CHCl_3_ and DMF and placed in quartz cuvettes of 10 mm path length. Solid state measurements were carried out for thin films spin coated on quartz plates from solutions in chloroform (c_Py_ = 10^−6^ mol/L).

#### 2.2.10. Fluorescence Energy Transfer

Experiments with dye additives (Nile Red, Coumarin 6) were carried out for a chosen amount (1–200 μL) of dye solution (10^−3^ mol/L, THF for triazole derivatives or DMF for amide derivatives) added to the solution (3 mL) of LPSQ/POSS-triazole-Py in THF or LPSQ/POSS-amide-Py in DMF at concentration 10^−6^ mol/L of Py groups.

#### 2.2.11. Detection of Metal Cations

For measurements with metal cations (Zn (II), Ca (II), Pb (II), Hg (II), Cu (II), Co (II), Ag (I), Fe (III), Mg (II)), a chosen amount (1–200 μL) of appropriate perchlorate (V) salt (10^−4^ mol/L, in THF for triazole derivatives or in DMF for amide derivatives) was added to the solution (3 mL) of LPSQ/POSS-triazole-Py in THF or LPSQ/POSS-amide-Py in DMF at concentration 10^−6^ mol/L of Py groups.

## 3. Results and Discussion

### 3.1. Synthesis, Structure and Thermal Properties of Fluorescent LPSQ

**Scheme 1:**
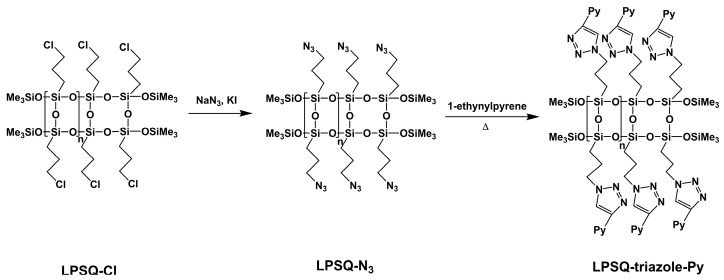
Synthesis of LPSQ-triazole-Py via copper(I)-catalyzed azide-alkyne cycloaddition of 1-ethynylpyrene to LPSQ-N_3_ (obtained from LPSQ-Cl precursor).

LPSQ are linear counterparts of the well-known polyhedral oligomeric silsesquioxanes (POSS), but due to their polymeric nature and highly ordered double-chain structures, they exhibit completely different physicochemical properties (e.g., solubility, thermal stability) [32,38,39,40]. LPSQ with a highly regular backbone can be prepared by polycondensation of cyclotetrasiloxane-2,4,6,8-tetraols [31] or step-wise coupling polycondensation [33]. The latter method was used for the preparation of polymeric precursors—poly(3-chloropropylsilsesquioxanes) (LPSQ-Cl; M_n_ = 1400 g/mol, M_w_ = 1700 g/mol, PDI = 1.3)—used in this study. LPSQ-Cl were converted into poly(3-azidopropylsilsesquioxanes) (LPSQ-N_3_) that were subsequently grafted with 1-ethynylpyrene via copper(I)-catalyzed azide-alkyne cycloaddition (Scheme 1). Polyhedral oligomeric silsesquioxanes were modified in a similar way.

**Figure 1:**
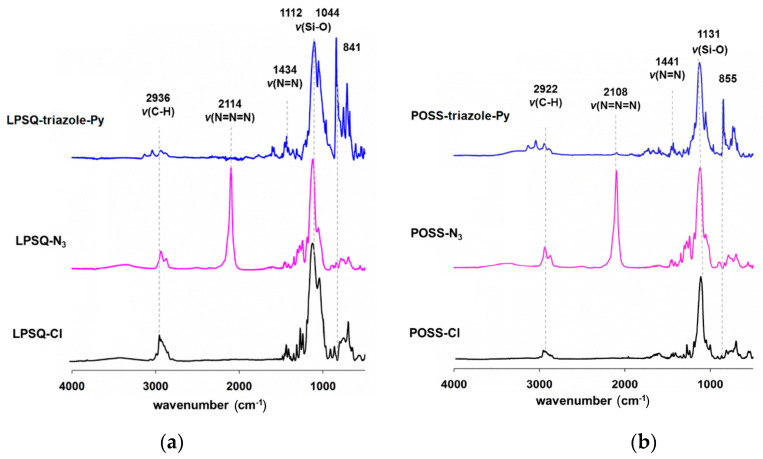
FTIR spectra plotted in absorbance mode (arbitrary units) of (**a**) LPSQ-triazole-Py and (**b**) POSS-triazole-Py along with spectra of the intermediate products.

The reaction progress was monitored by FTIR spectroscopy. The assignment of IR characteristic bands is presented in Figure 1 and Table S3. The synthesis was carried out until the complete conversion of –CH_2_CH_2_CH_2_Cl groups into –CH_2_CH_2_CH_2_N_3_ and then into –CH_2_CH_2_CH_2_–triazole. In the first step, disappearance of the band characteristic of stretching vibration of 3-chloropropyl groups ν(C–Cl) (~700 cm^−1^) was observed with simultaneous appearance of a new vibration typical for azide groups ν(N=N=N) (~2100 cm^−1^).

During the conversion of octahedral POSS-Cl into POSS-N_3,_ a reorganization of siloxane bonds was noted. A stable mixture of polyhedral oligomeric silsesquioxanes (octahedral POSS-T_8_-N_3_, decahedral POSS-T_10_-N_3_ and dodecahedral POSS-T_12_-N_3_) at 1:2:1 molar ratio was formed (Figure S3). The redistribution was reported before by Ervithayasuporn et al. [41]. The synthesis involves the use of a basic reagent (sodium azide, three-fold molar excess with respect to –CH_2_CH_2_CH_2_Cl groups) and is carried out in a solvent of high polarity (DMF). Under the applied reaction conditions, NaN_3_ plays a dual role of a source of azide groups and a nucleophile that induces cage rearrangement. It must be stressed that exactly the same composition of products was obtained if the reaction was carried out at [NaN_3_]_0_/[Cl]_0_ = 1/8. The obtained mixture of polyhedral azides was used for the further studies. The organization of side substituents in linear ladder structure differs significantly from those of any of the obtained polyhedral silsesquioxanes, and thus, we did not separate the POSS components of the mixture. LPSQ-Cl suffered similar redistribution of siloxane bonds induced by NaN_3_. The SEC diagram (Figure S2) of the crude reaction product (M_n_ = 6100 g/mol, M_w_ = 18,500 g/mol, PDI = 3.0) shows an increase in polydispersity with regard to LPSQ-Cl. Precipitation of the obtained material resulted in a product fraction (Y = 76%) of a narrow M_w_/M_n_ (PDI = 1.3) and molecular weight M_n_ of 1900 g/mol (Table S2). The purified product was used for further studies. During the Hüisgen coupling step, the conversion of azide groups was confirmed by disappearance of the ν(N=N=N) band in FTIR spectra. At the same time, vibration modes typical for a triazole ring—ν(N=N) (1434 cm^−1^) and ν(C=C) (1550 cm^−1^) appeared. The structures of LPSQ-triazole-Py and POSS-triazole-Py were confirmed with ^1^H, ^13^C and ^29^Si NMR (in solution and solid state) (Supplementary Information).

**Scheme 2:**
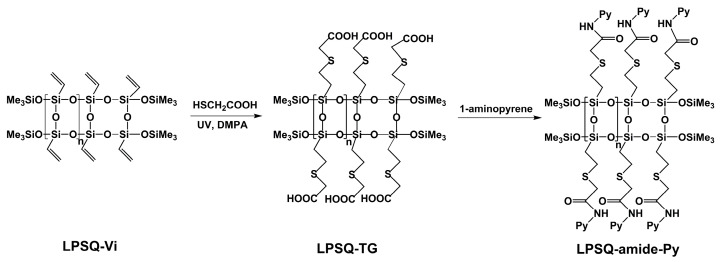
Synthesis of LPSQ-amide-Py via condensation of carboxylic groups of LPSQ-TG and 1-aminopyrene.

LPSQ bearing vinyl groups (LPSQ-Vi; M_n_ = 1000 g/mol, PDI = 1.2) was used as the precursor for macromolecules with Py chromophores grafted through linkers containing amide groups (Scheme 2). It was converted into poly[2-(carboxymethylthio)-ethylsilsesquioxane] (LPSQ-TG) by photoinitiated addition of thioglycolic acid to the side vinyl groups, as we have previously described [32]. The addition occurred regioselectively with formation of the anti-Markovnikov product. LPSQ-amide-Py was obtained by condensation of the side –COOH groups of LPSQ-TG with 1-aminopyrene molecules. The solubility of linear oligomers with all side chains containing amide bond (LPSQ-amide100-Py) was low owing to strong interchain interactions (formation of hydrogen bonds connecting amide linkers, analogously to those observed in polyamides [42]) and attractive π-π contacts between Py substituents. Due to the structural differences, the octa-substituted octahedral analogue (POSS-amide-Py) was very soluble in THF and DMF. To improve solubility of LPSQ bearing amide-Py groups and enable studies on their optical properties, soluble LPSQ-amide75-Py and LPSQ-amide50-Py, containing, respectively, 75 and 50% of Py substituted side chains, were prepared under the same synthetic protocol (Table S1).

**Figure 2:**
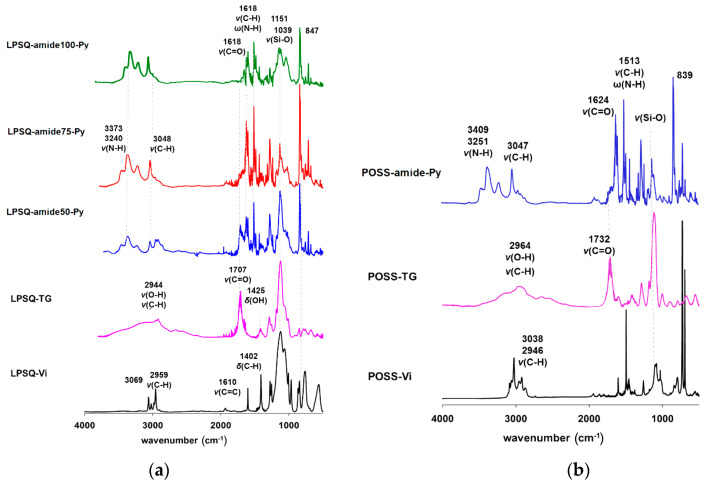
ATR-FTIR spectra plotted in absorbance mode (arbitrary units) of (**a**) LPSQ-amide-Py and (**b**) POSS-amide-Py along with spectra of the intermediate products.

The reaction progress was monitored by FTIR and it was carried out until the planned conversion of carboxylic groups into amides occurred. The assignment of IR characteristic bands is presented in Figure 2 and Table S3. Amide bond formation was confirmed by disappearance of the vibration band typical for ν(C=O) in carboxylic acids (1750–1700 cm^−1^) and formation of new bands characteristic of amides: ν(C(O)N) (1700–1600 cm^−1^) and ν(C-N) in combination with δ(N-H) (1600–1500 cm^−1^). The amount of amide groups in side chains influenced the position of vibrational bands. Increasing the ratio of amide groups led to the shift of ν(C(O)N) mode toward lower wavenumbers (from 1640 cm^−1^ for LPSQ-amide50-Py to 1618 cm^−1^ for LPSQ-amide100-Py). A similar position of the ν(C(O)N) band was found for POSS-amide-Py (1624 cm^−1^). All the obtained materials were characterized with ^1^H, ^13^C and ^29^Si NMR spectroscopy (in solution and in solid state) (Supplementary Information).

**Figure 3:**
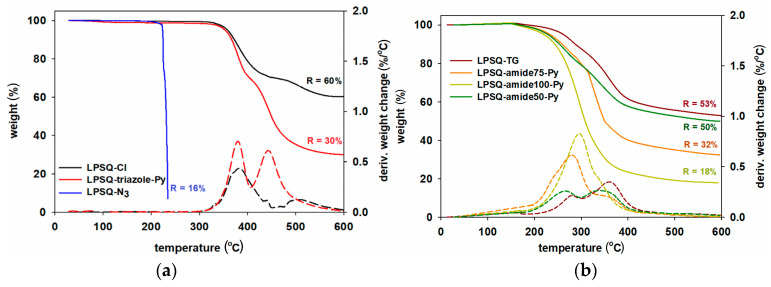
Thermogravimetric analysis of (**a**) LPSQ-triazole-Py and (**b**) LPSQ-amides-Py (N_2_ atmosphere, 10 °C/min).

**Figure 4:**
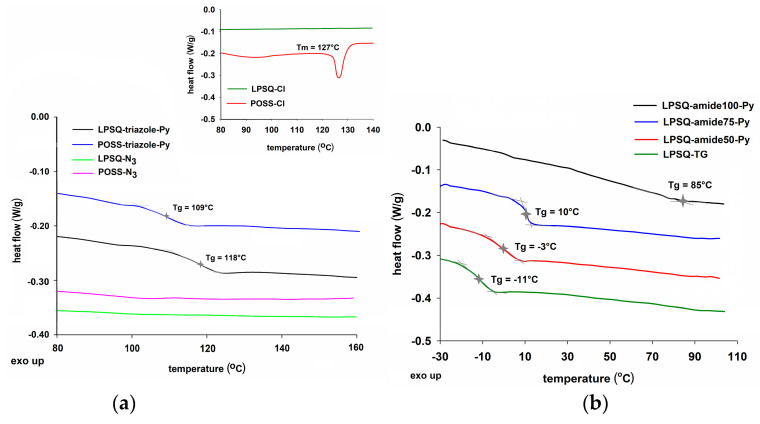
Thermal analysis of (**a**) LPSQ and POSS-triazole-Py (inset: LPSQ-Cl and POSS-Cl) (**b**) LPSQ-amide-Py. DSC traces recorded at a heating rate of 10 °C/min (3rd run).

Thermal characteristics of the obtained materials were assessed with TGA and DSC techniques (Figure 3, Figure 4 and Figure S4). Thermal stability of both triazole-containing silsesquioxanes increased in comparison to their azide precursors that underwent rapid sublimation at temperature > 235 °C. Azides are known to be thermosensitive and possible explosives. Their stability depends on their atom composition ((N_C_+N_O_)/N_N_ ≥ 3, where N—number of atoms) [43]. Organic alkyl azides typically start to decompose at 180–200 °C [44]. Octakis(3-azidopropyl)octasilsesquioxanes were reported to be thermally stable up to 237 °C [45], which is consistent with our results. We ascribed the observed similar thermal stability of POSS-N_3_ and LPSQ-N_3_ to the similar atom composition of these materials. LPSQ-triazole-Py and POSS-triazole-Py were thermally stable up to ∼350 °C in N_2_ atmosphere (5% weight loss temperature (T_5%_) = 353 °C and 352 °C for LPSQ and POSS, respectively) (Figure 3a and Figure S4a). A characteristic two step decomposition was observed for the triazole-silsesquioxanes and their 3-chloropropyl precursors. The two main decomposition stages of LPSQ-Cl leading to the high char yield (60%) may be ascribed to the partial degradation of the organic moieties and then scission of Si-C bonds at T >450 °C, analogously to those reported for other POSS [46,47]. The first decomposition step of LPSQ-triazole-Py (T_d1_ = 380 °C; 29% wt decrease) occurred in the temperature range similar to T_d1_ of LPSQ-Cl. The char left after the second stage of weight loss (T_d_ = 443 °C; 39% wt decrease) is larger than that calculated for the inorganic residue formed by cleavage of Si–C bonds. It suggests recombination of structural segments during the thermolysis and formation of a cross-linked material. This pathway may be supported by a larger amount of the char remaining at 600 °C after the decomposition of LPSQ-triazole-Py, while two-step T_d5_ were similar (Table 1).

Materials with amide-Py groups were less thermally stable than LPSQ-TG. The increasing amount of side amide groups corroborated the observed decrease in T_5%_ (Table 2). The weight loss and the rate of decomposition (V_d_) during the first decomposition step (T_d1_ = 265–300 °C) was higher for materials containing more amide-Py groups (from 20% wt decrease for LPSQ-amide50-Py to 78% for LPSQ-amide100-Py) and the char residue at 600 °C (Figure 3b, Table 2). The opposite situation was observed for the second mass loss stage (T_d2_ = 385–470 °C with weight loss from 30% for LPSQ-amide50-Py to 4% for LPSQ-amide100-Py). For POSS-triazole-Py, only one decomposition step was observed (Table 2, Figure S4a), and its T_d_ was similar to T_d1_ for LPSQ-amide100-Py (303 °C with 84% wt decrease). The lower thermal stability of LPSQ-amide-Py may be explained by decarboxylation of side chains. Such phenomena were observed for carboxylate-functionalized polymers at 250–370 °C [48]. It corresponds both to the observed T_d1_ (260–300 °C) and the extent of the process illustrated by the peak at T_d_ = 360–380 °C.

LPSQ-triazole-Py and LPSQ-amide-Py did not show any mesomorphic properties within the studied temperature range. Devitrification was noted only for LPSQ-triazole-Py and POSS-triazole-Py (T_g_ = 118 °C and 109 °C, respectively, Figure 4a). POSS-triazole-Py was prepared using POSS-N_3_ consisting of a mixture of octa-, deca- and dodecahedral species. It is known that physical properties of silsesquioxane cages are determined by the degree of symmetry at the molecular level [49]. POSS systems of different size can form crystalline materials—e.g., pure POSS-T_8_-N_3_ is crystalline, but a mixture of octa-, deca- and dodecasilsesquioxanes is amorphous [41]. Moreover, higher flexibility of Si–O–Si bonds in larger polyhedral cages may also be the cause of the lower crystallinity of these compounds [49]. Highly symmetrical octahedral silsesquioxanes are crystalline (e.g., octakis(3-propyl methacrylate)octasilsesquioxane [49], octakis(3-chloropropyl)octasilsesquioxane [50], octakis(para-nitrobenzene)octasilsesquioxane [51]) but deca- and dodecahedral silsesquioxanes may behave like polymeric materials (e.g., exhibit clear devitrification transitions) [49,51]. 

LPSQ-TG is a viscous liquid that undergoes devitrification at −11 °C. Grafting 1-aminopyrene in the side groups reduced flexibility of macromolecules, and T_g_ of the obtained LPSQ-amide-Py were found at higher temperatures (Figure 4b). The higher the pyrene moieties grafted along the polymer chains, the higher the observed T_g_ (−3 °C for LPSQ-amide50-Py; 10 °C for LPSQ-amide75-Py). Glass transition was barely visible for LPSQ-amide100-Py (about 85 °C). Octahedral POSS-amide-Py exhibited not only melting at around T_m_ = 104 °C but also a glass transition (T_g_ = 3 °C) (Figure S5). It may be explained by adjustment of more flexible segments in the crystal structure, analogously to that observed in macromolecular systems [52,53,54].

### 3.2. Organization of LPSQ and POSS Materials in Solid State

**Figure 5:**
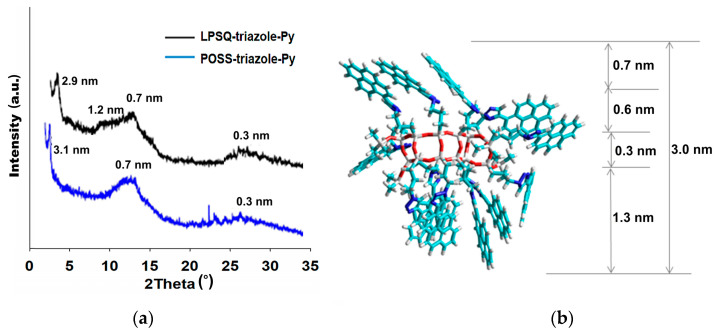
(**a**) WAXS diffractograms of thin films of LPSQ and POSS-triazole-Py. (**b**) HyperChem calculated structure for LPSQ-triazole-Py.

**Figure 6:**
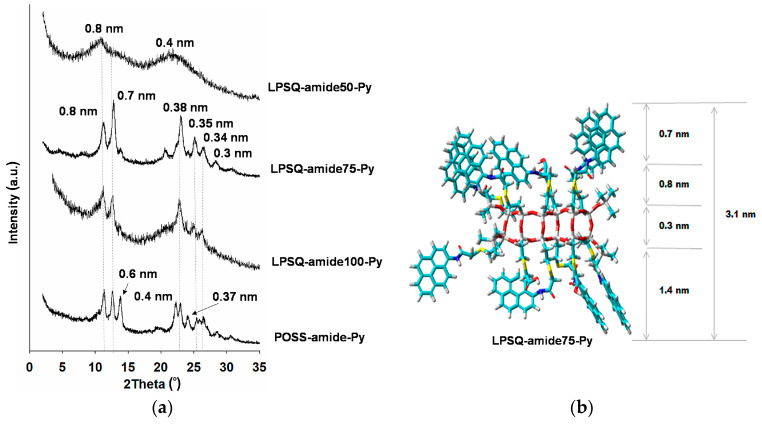
(**a**) WAXS diffractograms of thin films of LPSQ-amide-Py and POSS-amide-Py cast on silicon plates from DMF solutions; (**b**) HyperChem [37] calculated structure for LPSQ-amide75-Py.

Good solubility of LPSQ-triazole-Py, LPSQ-amide50-Py and LPSQ-amide75-Py allowed for the preparation of thin films on silicon supports. Solid state organization was studied by wide angle X-ray scattering analysis (WAXS) (Figure 5 and Figure 6). WAXS data revealed differences in the structure of LPSQ, POSS and silane-triazole-Py. The respective diffractograms of LPSQ and POSS show that the materials are amorphous, and three broad peaks with maxima corresponding to d = 3.0, 1.2–0.7 and 0.3 nm (2θ = 2.9°, 6.7–12.6° and 28°) can be ascribed to the material organization in the solid state (Figure 5). Such wide diffraction peaks are typical for ladder-like silsesquioxanes [55]. Ladder-like silsesquioxanes can form single crystals only when they have a defined structure, such as the tricyclic ladder-like 1,3,5,5,7,7,9,11,13,13,15,15-*cis-cisoid-cis*-dodecaisopropyltricyclo[9.5.I.I3,9]octasiloxane [56]. POSS-triazole-Py was a mixture of polyhedral species, and thus, it did not crystallize. The peak with the maximum at around 2θ = 28° (0.3 nm) may be correlated with Si–O–Si bonds. According to our HyperChem calculations, signals found for LPSQ-triazole-Py at around 3 and 1.2 nm (2θ = 2.9° and 6.7°) may correspond to the ladder width and approximate size of the side group, respectively. Differences between diffraction patterns of LPSQ and POSS result from different packing of the substituents around the inorganic core and different organization of pyrene groups in the solid state. WAXS analysis of silane-triazole-Py indicate that the material is partially crystalline (Figure S6a). A set of sharp diffraction peaks with maxima corresponding to d = 2.8, 1.5 and 0.75 nm (2θ = 3.1°, 6° and 12.8°) appeared. The signal at 2θ = 6° (d = 1.5 nm) may be assigned to the size of silane-triazole-Py molecules, and the peak around d = 2.8 nm (2θ = 3°) may be connected with the formation of π-stacks due to the π-π interactions between aromatic rings [57,58].

**Figure 7:**
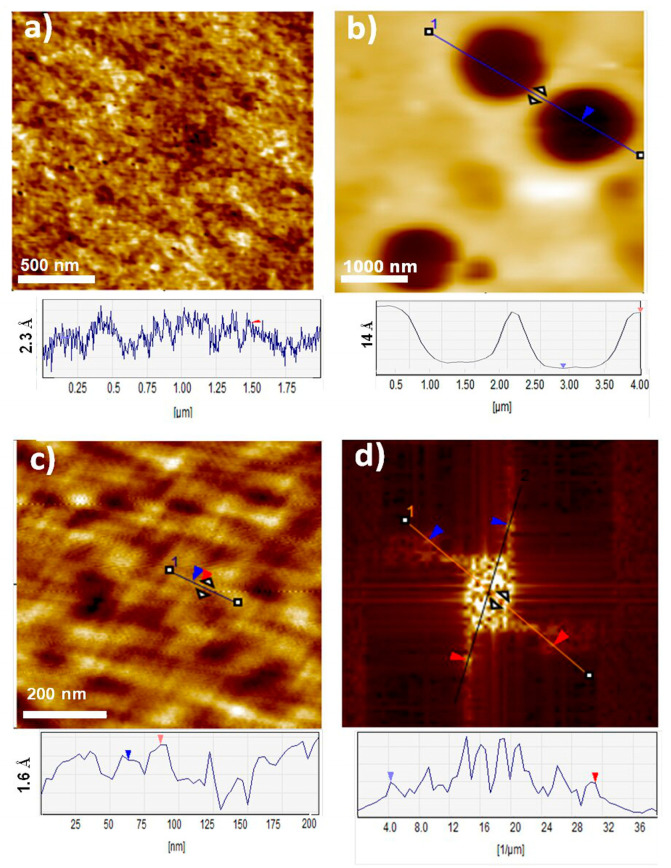
Atomic force microscopy height images and cross-sectional analysis of (**a**) LPSQ-triazole-Py; (**b**) POSS-triazole-Py thin films cast on silicon supports; (**c**) LPSQ-triazole-Py image after (**d**) FFT spectrum analysis.

The surface structure of thin films of LPSQ, POSS and silane-triazole was analyzed with AFM (Figure 7 and Figure S6b). The thin films covered the support completely. Layers formed by LPSQ-triazole-Py exhibited high regularity. Macromolecules were arranged in an oriented manner with an average distance between lamellae around 24 nm. The periodicity was confirmed by Fast Fourier Transform (FFT) of the AFM image that is often used to eliminate periodic noise from AFM images with repetitive patterns [59,60,61]. The periodicity can be connected with strong interchain interactions between pyrene molecules in the side chains. The film made of POSS-triazole-Py had an irregular structure, indicating formation of agglomerates. A similar difference in the structure of thin films was reported by us for LPSQ-Py and POSS-Py [30]. Silane-triazole-Py formed flat films of constant thickness (2.2 nm) (Figure S6b).

**Figure 8:**
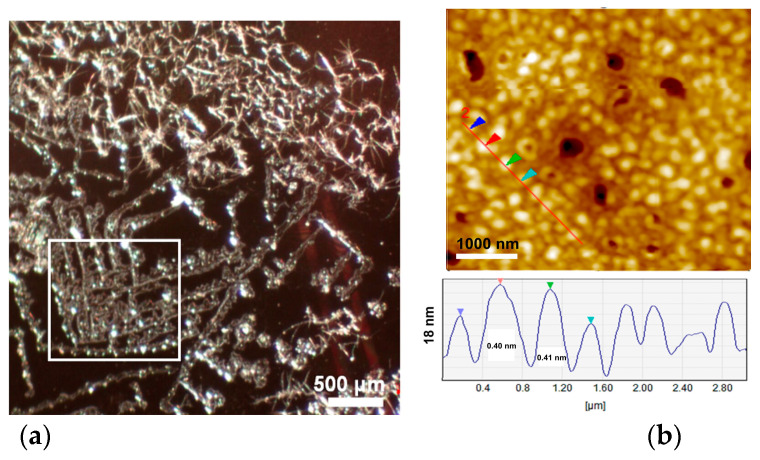
(**a**) Reflected light micrographs and (**b**) atomic force microscopy image and cross-section of LPSQ-amide75-Py crystals. AFM image shows the top of a typical crystal surface in the area marked with the white frame.

Thin films of LPSQ and POSS-amide-Py were prepared by drop casting of the respective DMF solutions on silicon supports. Reflected light microscopy images of LPSQ-amide75-Py revealed formation of fiber-like crystalline structures (Figure 8a). AFM analysis of the top layer of crystals revealed a certain periodicity (Figure 8b). Formation of thin twisted fibers of helical structure was also reported for LPSQ-Py [30]. LPSQ-amide50-Py and POSS-amide-Py formed large needle-like structures (length 150–200 μm) (Figure S7). This can be explained by aggregation of macromolecules during slow solvent evaporation resulting in the formation of well-organized crystals. In the case of POSS-amide-Py, the needle-like structures were present on the top of a crystalline base layer that covered the support. The morphology of amide containing silsesquioxanes was also studied by X-ray diffraction (Figure 6). Differences between diffraction patterns of LPSQ and POSS-amide-Py illustrate different organization of side groups in the solid state. This may be related to the amount of hydrogen bonds between side chains. The content of amide bonds in LPSQ-amide50-Py is too low to interconnect the side chains by hydrogen bonds. This product is amorphous, and its WAXS analysis exhibits only two wide peaks with maxima around 0.8 and 0.4 nm (2θ = 11° and 22°), that are typical for linear oligo(silsesquioxanes) with simple alkyl or aryl groups. Diffractograms of other products indicate their partial crystallinity. Analyses revealed multiple peaks corresponding to interplanar spacing d = 0.8, 0.7, 0.38, 0.35, 0.34 and 0.30 nm (2θ = 11°, 12.6°, 23.2°, 25.8°, 26.5° and 28°) (Figure 6). Peaks around 2θ = 11° (d = 0.8 nm) correspond to the length of linkers between pyrene molecules and LPSQ main chain or POSS core. Peaks around 2θ = 12.6° (d = 0.7 nm) can be associated with the size of pyrene moieties or distance between adjacent side substituents. Signals in the range 0.34–0.38 nm (2θ = 23.2–26.5°) might be related to the interplanar π-π distance between Py moieties [57,62]. LPSQ-amide75-Py contains an optimal amount of amide groups to obtain a good arrangement of side substituents for association by both hydrogen bonds and π-π interactions. The high degree of regularity in thin layers allowed us to obtain a diffractogram which shows intense peaks associated with the structure of LPSQ-amide75-Py. As could be expected, almost all side chains in LPSQ-amide100-Py were associated by the formation of hydrogen bonds. It significantly rigidified the entire macromolecule. A tight network of hydrogen bonds between the side substituents was already present in solutions used for the preparation of thin films that were analyzed with WAXS. The high content of side substituents with high steric hindrance, the presence of hydrogen bonds and π-π interactions leave less free space for an effective arrangement of all the side substituents. All peaks characteristic for LPSQ-amide-Py are present, but due to the lower degree of order, their intensity is quite low. WAXS analysis of POSS-amide-Py exhibited additional peaks corresponding to d = 0.6, 0.4 and around 0.37 nm (2θ = 14.7°, 22° and 23.8°).

### 3.3. Optical and Photophysical Properties—Comparison of LPSQ/POSS-Triazole and LPSQ-POSS-Amide

**Figure 9:**
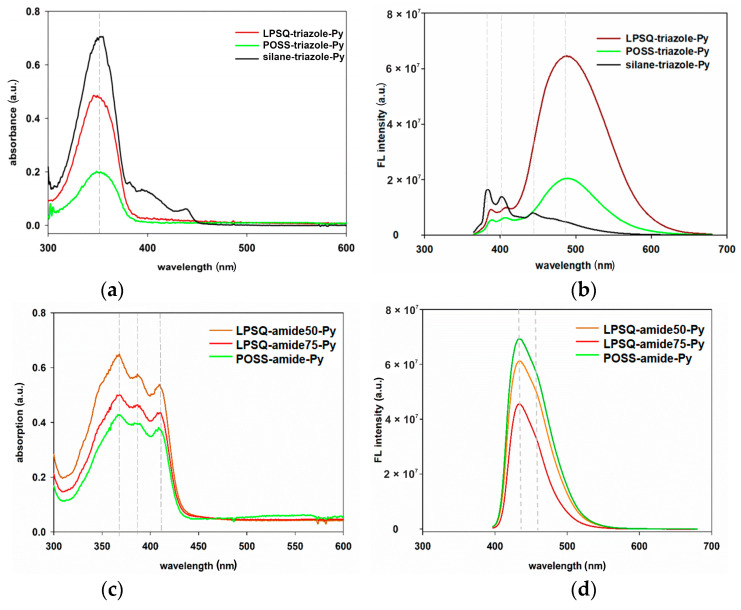
Comparison of (**a**) absorption and (**b**) FL emission spectra (λ_ex_ = 350 nm) of LPSQ, POSS, silane-triazole (c_Py_ = 10^−6^ mol/L in THF), (**c**) absorption and (**d**) FL emission spectra (λ_ex_ = 387 nm) of LPSQ-amide50-Py, LPSQ-amide75-Py and POSS-amide-Py (c_Py_ = 10^−6^ mol/L in DMF).

Optical and photophysical properties of the prepared LPSQ and POSS with pyrene moieties in side chains were studied at room temperature with UV–Vis and fluorescence spectroscopy in diluted solutions (c = 10^−8^–10^−3^ mol/L of Py units) and in the solid state. Both LPSQ and POSS bearing pyrene moieties in their side chains did not have clear-structured absorption bands—typical for small molecules. The absorption spectra of LPSQ and POSS are similar and consist of a single, wide band with only one maximum for LPSQ/POSS-triazole-Py (Figure 9a and Figure S8) and three maxima for LPSQ/POSS-amide-Py (Figure 9c and Figure S9). Such broad absorption bands suggest pre-association of chromophores in the ground state and the presence of self-assembled structures even in extremely diluted solutions (c_Py_ = 10^−8^ mol/L) [63].

**Figure 10:**
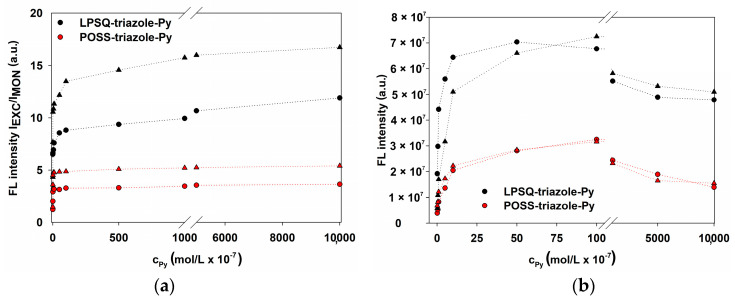
Comparison of fluorescence emission intensity (**a**) ratio I_EXC_/I_MON_ and (**b**) I_EXC_ as a function of pyrene groups concentration (● in THF, ▲ in CHCl_3_; black for LPSQ, red for POSS).

The steady-state fluorescence measurements were carried out using the excitation wavelengths of the S_0_ → S_2_ transitions (387 nm for solutions of LPSQ/POSS-amide-Py derivatives in DMF and 350 nm for solutions of LPSQ/POSS-triazole-Py in CHCl_3_ and THF). The structure of silsesquioxanes has a strong effect on their fluorescence (Figure 9b,d, Figure S9 and Figure 10). FL spectra of dilute solutions of LPSQ and POSS-triazole-Py (both in THF and CHCl_3_) exhibited almost exclusively a bright green excimer emission band with a maximum around 487 nm. In contrast, FL emission spectra obtained for the model silane-triazole-Py consist mostly of strong pyrene monomer emission and almost did not exhibit the excimer emission, even at c_Py_ = 10^−3^ mol/L. The presence of T_8_, T_10_ and T_12_ in the POSS-triazole-Py should not influence optical properties of the studied material. The three-dimensional structure of POSS, regardless of their size, does not facilitate interactions between Py substituents. The side chains have much more freedom to move around the POSS core, which leads to the predominant influence of the π-π interactions between Py moieties.

Traces of monomeric vibronic bands could be observed in all solutions of LPSQ and POSS-triazole-Py in (THF and CHCl_3_). The ratio of excimer (I_EXC_) (λ_EXC_ = 490 nm) and monomer (I_MON_) (λ_MON_ = 407 nm) FL emission intensity is 3–4 times higher for LPSQ-triazole-Py (Figure 10a), which can be ascribed to different geometry of the inorganic structures and associated orientation of the pyrene moieties. High intensity of the excimer emission band in the most diluted solutions of LPSQ-triazole-Py points to its intramolecular nature due to the congestion of chromophores along the polymer chain. The increase in Py concentration resulted in a slight bathochromic shift of λ_EXC_ from 487 nm to 490 nm.

FL emission intensity of LPSQ derivative is more than twice as high as that of POSS (Figure 10b and Figure S10). FL quenching occurred at a similar concentration for both silsesquioxanes (at c_Py_ ≥ 5 × 10^−5^ mol/L). No solvent effect was observed concerning the intensity and shape of FL emission bands of LPSQ and POSS-triazole-Py.

**Figure 11:**
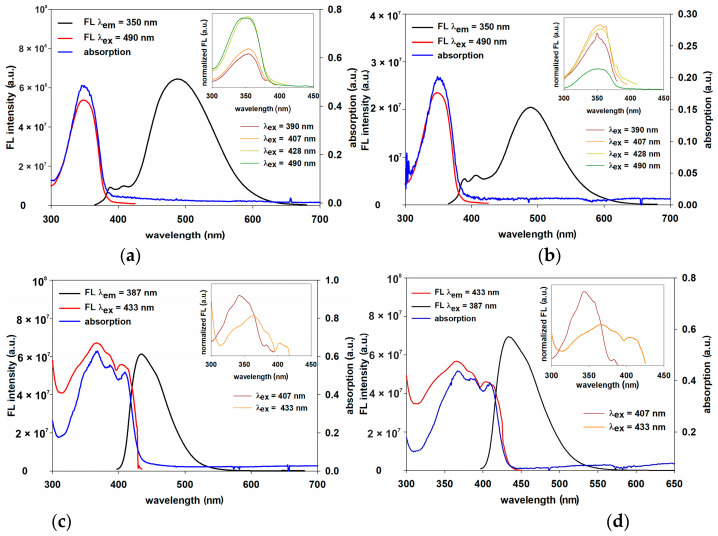
Emission and excitation fluorescence spectra with absorbance spectra of (**a**) LPSQ-triazole-Py, (**b**) POSS-triazole-Py (in THF) and (**c**) LPSQ-amide50-Py, (**d**) POSS-amide-Py (in DMF). All measurements for solutions at c_Py_ = 10^−6^ mol/L.

The excitation spectra of LPSQ and POSS-triazole-Py in THF were recorded at the emission wavelength of the excimer (λ_EXC_ = 490 nm) (Figure 11a,b). The observed degree of correlation with the respective absorption spectra was almost identical for LPSQ and POSS. Excitation spectra were also recorded at λ = 390, 407, 428 and 490 nm (Figure 11a,b). Their overlap was exact for LPSQ-triazole-Py. This result indicates the dynamic nature of the excimer [64,65]. Similar results were obtained for POSS-triazole-Py.

**Figure 12:**
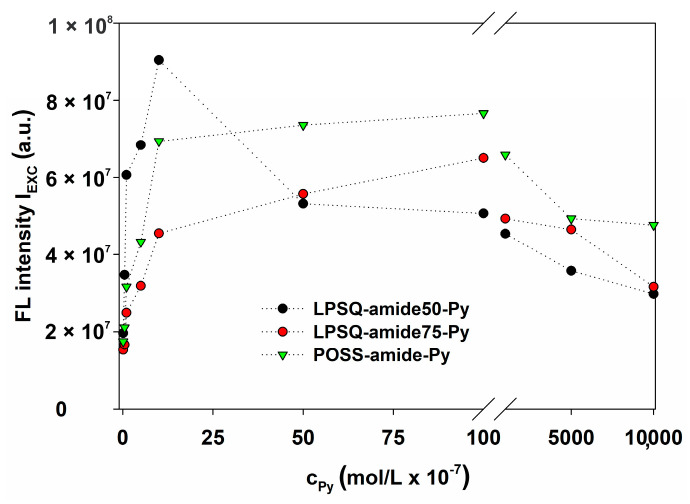
Changes in fluorescence excimer intensity as a function of pyrene moieties concentration (λ_ex_ = 430 nm).

FL spectra of dilute solutions of LPSQ and POSS-amide-Py in DMF exhibited exclusively a bright emission band with a maximum at 433 nm (Figure 9d and Figure S9). High intensity of this emission band, even in the most diluted solutions, suggests the intramolecular nature of excimers due to the congestion of chromophores along the polymer chain. The shape of FL emission bands of all materials bearing amide groups in their side chains is very similar. Spectra with a maximum of FL emission intensity at ~430 nm was previously reported for various pyrene-1-carboxamides [66,67]. The decrease in fluorescence intensity with the increase in concentration in solution occurred more rapidly for LPSQ-amide50-Py (c_Py_ ≥ 10^−6^ mol/L) than for LPSQ-amide75-Py and POSS-amide-Py (c_Py_ ≥ 5 × 10^−5^ mol/L) (Figure 12). this can be explained by a more pronounced influence of intermolecular interactions for LPSQ-amide50-Py.

The excitation spectra of LPSQ and POSS-amide-Py in DMF were recorded at the wavelength λ_em_ = 433 nm that responded to the maximum of FL emission intensity (Figure 11c,d and Figure S11). Excitation spectra were also recorded in DMF at the emission wavelength of 407 nm. The observed degree of correlation with the respective absorption spectra was very similar. They do not overlap which suggests that observed excimers for LPSQ and POSS-amide-Py are static in nature and their formation is not dependent on the diffusion or the solution concentration [68].

**Figure 13:**
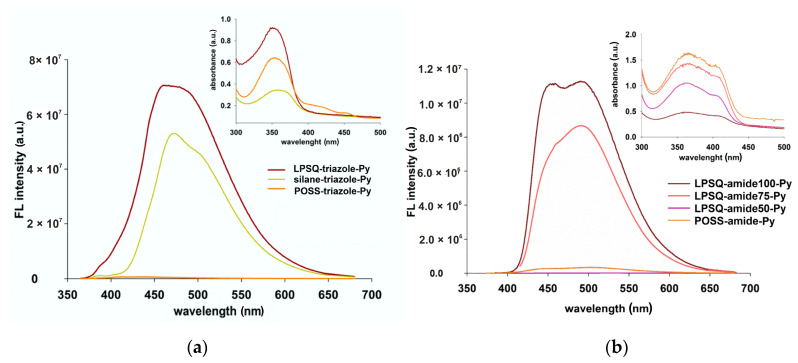
FL and absorption spectra for thin films of LPSQ and POSS materials containing (**a**) triazole and (**b**) amide groups in side substituents.

Despite the structural differences, both LPSQ and POSS-triazole-Py are emissive in the solid state (Figure 13a). The much stronger intensity of LPSQ-triazole-Py FL emission than that of the POSS counterpart can be ascribed to the stronger interactions between pyrene moieties along the linear chains than around 3D inorganic cores of the polyhedral compound. The λ_max_ in the FL spectrum of LPSQ-triazole-Py is blue shifted in comparison to POSS-triazole-Py which illustrates the difference in supramolecular organization of Py moieties in the solid state for both materials. Differences in the FL emission λ_max_ were previously observed for LPSQ-Py and POSS-Py [30]. For the studied silsesquioxane derivatives containing amide groups, LPSQ-amide100-Py and LPSQ-amide75-Py exhibited the highest FL emission intensity (Figure 13b). The observed differences in the FL emission intensity of LPSQ-amide100-Py and POSS-amide-Py were similar to the FL emission intensity of LPSQ and POSS-triazole-Py. LPSQ-amide50-Py exhibited the lowest FL emission intensity (Figure 13b).

### 3.4. Energy Transfer with LPSQ-Triazole-Py and LPSQ-Amide-Py

The efficiency of fluorescence resonance energy transfer depends on the distance between donor and acceptor molecules and the overlap of the emission spectrum of the donor and the absorbance spectrum of the acceptor. It is thus important to choose spectrally suitable species to transfer energy from green-emitting pyrene excimers. Organic dyes such as 9-diethylamino-5-benzo[α]phenoxazinone (Nile Red, NR) and 3-(1,3-Benzothiazol-2-yl)-7-(diethylamino)-2H-chromen-2-one (Coumarin 6, Cou) are suitable for such artificial energy transfer systems [69,70,71,72].

**Figure 14:**
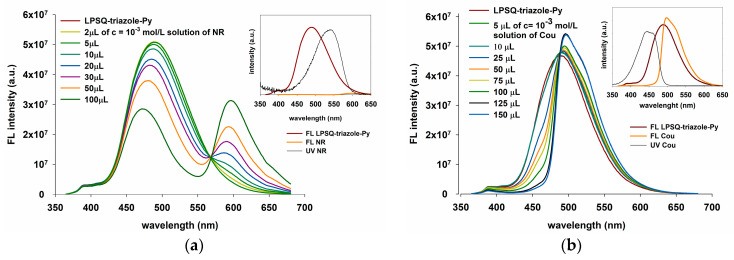
Energy transfer from LPSQ-triazole-Py to (**a**) Nile Red and (**b**) Coumarin 6 (insets: spectral overlap of LPSQ derivatives and dyes; measurements carried out as described in Materials and Methods).

**Figure 15:**
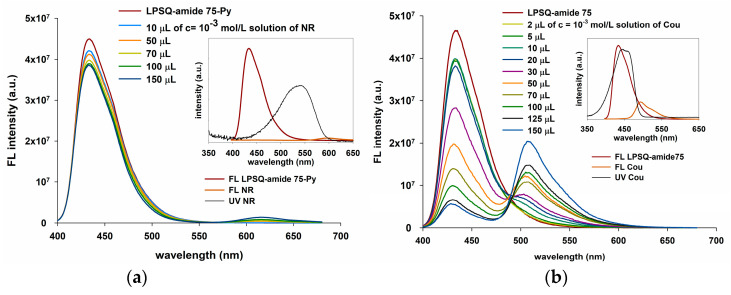
Energy transfer from LPSQ-amide75-Py to (**a**) Nile Red and (**b**) Coumarin 6 (insets: spectral overlap of LPSQ derivatives and dyes; measurements as described in Materials and Methods).

We found that macromolecules of LPSQ-triazole-Py were able to act as energy donors to red-emitting Nile Red (Figure 14a). As required for an efficient resonance transfer of energy, the emission of LPSQ-triazole-Py overlaps well with the absorption spectrum of NR in solution in CHCl_3_. Excitation at 350 nm involved mainly Py excimers, and the NR acceptor emission was observed with a decrease in the Py excimer emission due to fluorescence resonance energy transfer. A slight change in the shape of the Py excimer emission band was also noted with increasing NR concentration in the mixture. This suggests a selective fluorescence resonance energy transfer from a defined range of spectrally suitable excimers. The fluorescence resonance energy transfer was also studied from POSS-triazole-Py to NR (Figure S12a). The size of POSS should not affect EnT, so all tests were carried out on the prepared mixture of POSS-T_8_-N_3_, POSS-T_10_-N_3_ and POSS-T_12_-N_3_ (POSS-triazole-Py). Due to the small degree of overlapping between FL emission spectra of amide containing LPSQ and NR absorption bands, the energy transfer in this system occurred only to a very small extent (Figure 15a).

Coumarin 6 was also tested for the resonance energy transfer from Py excimers in LPSQ-triazole (Figure 14b), LPSQ-amide50 (Figure S13a) and LPSQ-amide75 (Figure 15b). The characteristic emission of Cou increased with quenching of the pyrene excimer band. The obtained results suggest the existence of a pathway for fluorescence resonance energy transfer from LPSQ-triazole-Py and LPSQ containing side amide groups to the acceptor dye. A decrease in FL emission intensity was observed for LPSQ-triazole-Py, LPSQ-amide50-Py and LPSQ-amide75-Py in the presence of Cou, but only for amide containing LPSQ, it was accompanied by an increase in Cou FL intensity. The degree of overlapping of FL emission bands of LPSQ-amide-Py and Cou was higher than that observed in the case of POSS-triazole-Py, but I_EXC_ of LPSQ-amide-Py was also around three times higher than that of Cou, when excited at λ = 387 nm. FL emission intensities of Cou and LPSQ-triazole-Py were very similar when excited at λ = 350 nm. In this case, the characteristic emission of Cou increased with quenching of the LPSQ-triazole-Py excimer band, but those two overlapped extensively and changes in the intensity of the former were not easy to notice. Different maxima of FL emission of Cou and LPSQ-amide-Py additionally facilitated the observation of fluorescence emission energy transfer.

### 3.5. Cation Sensing Ability of the Hybrid Silsesquioxanes

**Figure 16:**
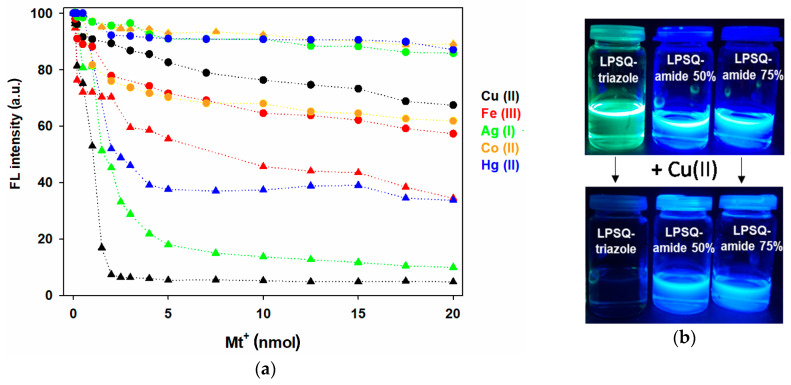
(**a**) Comparison of FL emission intensity of ▲—LPSQ-triazole-Py (in THF) and ●—LPSQ-amide50-Py (in DMF) in the presence of perchloric (VII) acid salts; (**b**) visible changes of FL emission intensity of LPSQ-triazole-Py, LPSQ-amide50-Py and LPSQ-amide75-Py in the presence of Cu(II) ions (n_Cu(II)_ = n_Py_).

The fluorescent nature of all the prepared materials was also explored for detection of paramagnetic [Cu(II), Fe(III), Co(II)] and diamagnetic [Ag(I), Hg(II), Mg(II), Ca(II), Pb(II), Zn(II)] cations. Metal ions influence properties of molecules containing heteroatoms and play an important role in many biological processes [72,73]. Interactions of paramagnetic cations with π electrons of pyrene moieties have previously been exploited in fluorescent sensing systems [74,75,76,77,78]. We conducted comparative studies on the sensitivity and selectivity of cation detection on the basis of the fluorescence response with an individual addition of diverse perchloric (VII) acid salts to solutions of LPSQ/POSS-amide-Py (in DMF) and LPSQ/POSS-triazole-Py (in THF) (Figure 16, Figures S14 and S15). The size of POSS and the content of prepared product mixture should not affect its optical properties in the presence of cations; all tests were carried out on the prepared mixture of POSS-T_8_-N_3_, POSS-T_10_-N_3_ and POSS-T_12_-N_3_ (POSS-triazole-Py).

The fluorescence intensity of LPSQ-amide50-Py, LPSQ-triazole-Py and POSS-triazole-Py noticeably decreased with the addition of paramagnetic cations: Cu(II) and Fe(III) and Co(II). Changes in FL emission intensity were also observed for triazole-containing materials in the presence of diamagnetic Ag(I) and Hg(II) ions. Sensing ability of pyrene derivatives toward Cu(II) and Fe(III) stems from, respectively, reverse-PET phenomenon [79,80] and electron transfer from the excited Py chromophores [81]. Py FL quenching in the presence of Ag(I) and Hg(II) can be explained by the heavy atom effect [79].

While the studied triazole-containing materials exhibited high sensing ability toward cations, the change in FL intensity for LPSQ-amide75-Py was influenced only slightly. This may be explained in terms of strong hydrogen bonds between the side substituents that make Py chromophores better packed and less accessible for metal cations. Indeed, LPSQ-amide50-Py detected the presence of Cu(II), Fe(III) and Co(II), although the effect was not large. Interestingly, POSS-amide-Py exhibited FL emission quenching only in the presence of Co(II) ions (Figure S15a,b). The highest sensitivity of POSS-amide-Py toward Co(II) cations can be explained by low steric hindrance and easy accessibility to amide groups in side chains around the POSS core. Cobalt is among the essential trace elements and plays a crucial role in biological systems [82], but it is toxic at a high level [83], and thus, sensitive methods of its detection are needed. Compounds containing amide, amine and carboxyl groups were proposed as possible molecular colorimetric or fluorescent probes based on photoinduced electron transfer (PET) or chelation-enhanced fluorescence due to their ability to coordinate Co(II) [84,85]. It may be expected that Co(II) is also complexed by amide bonds in neighboring side groups of POSS-amide-Py, but the sensing mechanism rather involves the excimer fluorescence quenching.

The difference in sensitivity of LPSQ-triazole-Py and LPSQ-amide50-Py is presented in Figure 16a. The photographs show dramatic fluorescence emission changes of LPSQ-triazole-Py in the presence of Cu(II) ions (under UV lamp). In contrast, almost no visible change in FL emission intensity of LPSQ-amide50-Py and LPSQ-amide75-Py was observed (Figure 16b). It is possible that sensitivity of materials was influenced by the properties of chosen solvent. A highly polar medium may strengthen π-π interactions between aromatic moieties and thus hinder M^n+^-π interactions [86]. DMF applied as a solvent for amide containing silsesquioxanes exhibits much higher polarity (ε = 38, D = 3.82) than THF (ε = 7.5, D = 1.75) used for studies with LPSQ/POSS-triazole-Py. This might affect the influence of metal cations on FL emission intensity.

Studies on the selectivity of FL emission quenching in the presence of mixtures of various cations appear very interesting; therefore, we intend to carry out such tests in the next stage of our research on new optically active materials.

## 4. Conclusions

Pyrene chromophores were grafted onto hybrid silsesquioxane macromolecules of various morphologies by means of Hüisgen coupling or amide bond formation. The unique architecture of linear, ladder-like poly(silsesquioxanes) bearing Py moieties in side chains favors the formation of intramolecular excimers, both in very diluted solutions and in the solid state. The observed optical properties depend on the structure of silsesquioxane framework (linear or polyhedral), spacing between chromophores, morphology of linkers between pyrene moieties and polymer backbone and restriction of their motion. The formation of hydrogen bonds between the spacers containing amide bonds and linking Py substituents to the inorganic backbone determined optical and physicochemical properties of LPSQ-amide-Py.

It was found that LPSQ-triazole-Py and LPSQ-amide-Py are capable of an efficient fluorescence resonance energy transfer to suitable red-emitting dyes. The prepared materials also exhibited interesting sensing ability toward paramagnetic [Cu(II), Fe(III), Co(II)] and diamagnetic [Ag(I), Hg(II)] metal cations. Good photo-optical properties and processability of hybrid LPSQ-R-Py make them promising materials for possible applications in optoelectronics and metal cations sensing devices.

## Figures and Tables

**Table 1:** Thermal degradation characteristics of LPSQ and POSS-triazole-Py and their precursors.

Sample	T_d1_ (°C)	T_d2_ (°C)	Rate (%∙min/°C)		T_5%_ (°C)	Residue (%)	Residue Calculated for SiO_2_ (%)
			V_1_	V_2_			
LPSQ-Cl	382.2	509.6	2.90	1.09	358	60.2	46.2
LPSQ-N_3_	–	–	–	–	223	16.2	44.1
LPSQ-triazole-Py	379.7	443.0	2.86	3.78	353	30.0	16.5
POSS-Cl	358.2	502.1	5.56	0.63	358	37.3	46.3
POSS-N_3_	–	–	–	–	235	12.7	44.1
POSS-triazole-Py	376.7	434.7	2.23	1.73	352	57.5	16.6
silane-triazole-Py	365.3	403.0	7.35	1.11	213	10.2	16.5

T_d_—peak temperature of weight loss derivative. V—rate of weight loss at major decomposition step. T_5%_—5% weight loss temperature. residue—char residue at 600 °C.

**Table 2:** The parameters of thermal degradation of LPSQ and POSS-amide-Py and their precursors.

Sample	T_d1_ (°C)	T_d2_ (°C)	Rate (%∙min/°C)		T_5%_ (°C)	Residue (%)	Residue Calculated for SiO_3/2_ (%)
			V_d1_	V_d2_			
LPSQ-TG	279.5	362.6	1.99	3.12	262	53.1	32.0
LPSQ-amide50-Py	264.8	386.9	1.89	2.53	229	49.8	19.2
LPSQ-amide75-Py	279.8	-	6.36	-	233	31.9	16.4
LPSQ-amide100-Py	298.6	467.0	7.52	0.25	220	18.2	14.3
POSS-TG	380.0	-	1.72	-	190	47.1	27.8
POSS-amide-Py	303.4	-	7.54	-	225	20.0	13.4

## Supplementary Materials

The following are available online at https://www.mdpi.com/1996-1944/13/20/4491/s1, Synthetic procedures, Table S1: Composition of reaction mixtures during preparation of LPSQ/POSS-amide-Py; Figure S1: ^29^Si NMR (CDCl_3_) spectra of POSS-Cl and POSS-N_3_; Figure S2: SEC analysis of LPSQ-N_3_; Table S2: Molecular weight estimation based on SEC and NMR (^1^H, ^29^Si) analysis of LPSQ-Cl and LPSQ-N_3_; Figure S3: ^29^Si NMR (CDCl_3_) spectra of LPSQ-Cl and LPSQ-N_3_; Figure S4: Thermogravimetric analysis of POSS-triazole-Py and POSS-amide-Py; Figure S5: DSC analysis of POSS-amide-Py; Table S3: IR band position assignments; Figure S6: AFM and WAXS analysis of silane-triazole-Py; Figure S7: Reflected light microscopy images of LPSQ-amide50-Py and POSS-amide-Py; Figure S8: Absorption spectra of LPSQ, POSS and silane-triazole-Py solutions in CHCl_3_ and THF; Figure S9: FL emission spectra of LPSQ and POSS-triazole-Py in CHCl_3_; Figure S10: Absorption spectra of LPSQ and POSS-amide-Py; Figure S11: Emission and excitation fluorescence spectra of silane-triazole-Py and POSS-amide-Py; Figure S12: Energy transfer from POSS-triazole-Py to Nile Red and Coumarin 6; Figure S13: Energy transfer from LPSQ-amide50-Py and POSS-amide-Py to Coumarin 6; Figure S14: Changes in FL emission intensity of LPSQ and POSS materials in the presence of metal cations; Figure S15: Changes in FL emission intensity of POSS-triazole-Py and POSS-amide-Py in the presence of cations.
