# Supplementary material for: Hybrid Fluorescent Poly(silsesquioxanes) with Amide- and Triazole-Containing Side Groups for Light Harvesting and Cation Sensing
Source: Materials (Basel). 2020 Oct 10;13(20):4491. doi: 10.3390/ma13204491 (PMC7600812; doi:10.3390/ma13204491)
Supplement: Supplementary file 1 [file materials-13-04491-s001.pdf]

materials

Supporting Information

Contents:

1. Synthetic procedures

- Preparation of LPSQ-TG and POSS-TG
- General procedure for the synthesis of LPSQ-amide-Py and POSS-amide-Py
- Synthesis of LPSQ-Cl and POSS-Cl
- Synthesis of LPSQ-N_3 and POSS-N_3:

Figure S1. ^{29}Si NMR ($CDCl_3$) spectra of POSS-Cl and POSS-N_3.

Figure S2. SEC analysis of LPSQ-N_3

Figure S3. ^{29}Si NMR ($CDCl_3$) spectra of LPSQ-Cl and LPSQ-N_3.

- Preparation of LPSQ-triazole-Py and POSS-triazole-Py
- Synthesis of (3-azidopropyl)trimethoxysilane (silane-triazole-Py)

2. Characterization

Figure S4. Thermogravimetric analysis of POSS-triazole-Py and POSS-amide-Py.

Figure S5. DSC analysis of POSS-amide-Py.

Table S3. IR band position assignments.

Figure S6. AFM and WAXS analysis of silane-triazole-Py.

Figure S7. Reflected light microscopy images of LPSQ-amide50-Py and POSS-amide-Py.

Figure S8. Absorption spectra of LPSQ, POSS and silane-triazole-Py solutions in $CHCl_3$ and THF.

Figure S9. Absorption spectra of LPSQ and POSS-amide-Py.

Figure S10. FL emission spectra of LPSQ and POSS-triazole-Py in $CHCl_3$.

Figure S11. Emission and excitation fluorescence spectra of silane-triazole-Py and POSS-amide-Py

Figure S12. Energy transfer from POSS-triazole-Py to Nile Red and Coumarin 6.

Figure S13. Energy transfer from LPSQ-amide50-Py and POSS-amide-Py to Coumarin 6.

Figure S14. Changes in FL emission intensity of LPSQ and POSS materials in the presence of metal cations.

Figure S15. Changes in FL emission intensity of POSS-triazole-Py and POSS-amide-Py in the presence of cations.

3. Literature

Synthetic procedures:

Preparation of LPSQ-TG and POSS-TG

Linear LPSQ-TG were prepared following literature procedure [1]. A viscous polymeric product was obtained with 88% yield (8 g).

^{1}H NMR (THF-d_8): 0.14 ppm ($OSiMe_3$), 1.0 ppm ($SiCH_2$), 2.75 (CH_2S), 3.1 ppm (SCH_2), 10.5 ppm (COOH); ^{13}C NMR (THF-d_8): 1.5 ppm ($OSiMe_3$), 12.7 ppm ($SiCH_2$), 24.8 ppm (CH_2S), 32.5 ppm (SCH_2); ^{29}Si NMR (THF-d_8): -70.7 ppm, 10.5 ppm

The polyhedral analogue POSS-TG was prepared in an analogous way to LPSQ-TG. DMPA (35 mg) was added to a solution of thioglycolic acid (0.45 g) and POSS-Vi (0.5 g) in dry THF (35 mL), placed in a quartz vessel. The mixture was irradiated for 40 min with UV light (350 nm). Volatiles were then removed under reduced pressure. The residue was dissolved in THF and precipitated into hexanes. The purification procedure was repeated thrice. The precipitate was dried under high vacuum at room temperature to a constant weight. A viscous product was obtained with 98% yield (1.1 g).

^{1}H NMR (THF-d_8): 1.0 ppm ($SiCH_2$), 2.73 (CH_2S), 3.6 ppm (SCH_2), 10.7 ppm (COOH); ^{13}C NMR (THF-d_8): 12.8 ppm ($SiCH_2$), 25.3 ppm (CH_2S), 32.4 ppm (SCH_2); 173.0 ppm (COOH), ^{29}Si NMR (THF-d_8): -68.5 ppm

General procedure for the synthesis of LPSQ-amide-Py and POSS-amide-Py

LPSQ-TG (or POSS-TG) dissolved in freshly distilled THF, were charged under argon into a Schlenk flask. 1-aminopyrene in toluene was added to the solution and the reaction mixture was stirred for 15 min at room temperature. SiO_2 was added as a heterogeneous, reusable catalyst for direct amidation of side carboxylic acid groups [2] and the mixture was heated at 100°C. The progress of amide bond formation reaction was monitored using FTIR following gradual disappearance of band characteristic for carboxyl group vibrations. Once the reaction was complete the reaction mixture was cooled down to room temperature, filtered and concentrated under reduced pressure. The residue was dissolved in CH_2Cl_2 and precipitated to methanol. The purification procedure was repeated thrice. The collected product was purified by washing with THF and then methanol. It was dried under high vacuum to constant weight. Product was obtained as a light-green solid. We have prepared linear poly(silsesquioxanes) with different molar contribution of side amide groups e.g. LPSQ-amide50-Py containing 50 mol% of amide groups and 50 mol% of carboxyl groups in side chains (Table S1).

Table. S1. Composition of reaction mixtures during preparation of LPSQ/POSS-amide-Py.

Reagent	LPSQ-Amide50-Py	LPSQ-Amide75-Py	LPSQ-Amide100-Py	POSS-Amide-Py
LPSQ-TG or POSS-TG*	2 g	2 g	1 g	0.5 g
1-aminopyrene	1.27 g	1.9 g	1.27 g	0.63 g
toluene	200 mL	200 mL	100 mL	40 mL
THF	40 mL	40 mL	20 mL	4 mL
SiO_2	0.65 g	0.65 g	0.32 g	0.2 g
reaction yield	66% (2.1 g)	68% (2.3 g)	75% (1.8 g)	35% (0.39 g)

* - POSS-TG was used for preparation of POSS-amide-Py

<u>LPSQ-amide100-Py:</u> ^{13}C HP Dec NMR: 1.2 ppm ($OSiMe_3$), 12.4 ppm ($SiCH_2$), 26.7 ppm (CH_2S), 43.4 ppm (SCH_2), 122.4 ppm (Ar); ^{29}Si HP Dec NMR: -71.7 ppm, 10.0 ppm

LPSQ-amide75-Py: ^{1}H NMR (CD_3OD): 0.1 ppm ($OSiMe_3$), 1.0 ppm ($SiCH_2$), 2.7 ppm (CH_2S), 3.3 ppm (SCH_2), 7.2 - 7.4 ppm, 8.0 ppm (Ar), 8.7 (NH); ^{13}C HP Dec NMR: 1.7 ppm ($OSiMe_3$), 13.5 ppm ($SiCH_2$), 38.0 ppm (CH_2S), 54.0 ppm (SCH_2), 104.1-114.1 ppm, 116.3 ppm, 122.3 ppm, 140.2 ppm (Ar); ^{29}Si HP Dec NMR: -70.5 ppm, 10.5 ppm

LPSQ-amide50-Py: ^{1}H NMR (CD_3OD): 0.1 ppm ($OSiMe_3$), 0.9 ppm ($SiCH_2$), 3.0 ppm (CH_2S), 3.8 ppm (SCH_2), 7.7 - 7.9 ppm (Ar), 8.7 (NH); ^{13}C HP Dec NMR: 0.8 ppm ($OSiMe_3$), 13.1 ppm ($SiCH_2$), 33.4 ppm (CH_2S), 37.6 ppm (SCH_2), 103.6 ppm, 113.1 ppm, 115.6 ppm, 119.6 ppm, 122.3 ppm, 124.7 ppm, 136.2 ppm, 141.2 ppm (Ar); ^{29}Si HP Dec NMR: -71.2 ppm, 11.0 ppm

POSS-amide-Py: ^{1}H NMR (CD_3OD): 1.1 ppm ($SiCH_2$), 3.1 ppm (CH_2S), 3.9 ppm (SCH_2), 7.7 - 8.1 ppm (Ar), 8.6 (NH);^{13}C NMR (CD_3OD): 25.6 ppm ($SiCH_2$), 30.7 ppm (CH_2S), 34.5 ppm (SCH_2), 114.0 ppm, 116.8 ppm, 120.2 ppm, 124.3 ppm, 132.2 ppm, 135.8 ppm, 140.8 ppm (Ar); ^{29}Si HP Dec NMR: -70.3 ppm

Synthesis of LPSQ-Cl and POSS-Cl

LPSQ-Cl were prepared following the literature procedure [3]. A viscous product (M_n = 1400 g/mol, PDI = 1.3) was obtained with 37% yield (13.7 g).

^{1}H NMR ($CDCl_3$): 0.13 ppm ($OSiMe_3$), 0.8 ppm ($SiCH_2$), 1.8 ppm (CH_2), 3.5 ppm (CH_2Cl); ^{13}C NMR ($CDCl_3$): 1.7 ppm ($OSiMe_3$), 9.7 ppm ($SiCH_2$), 26.4 ppm (CH_2), 47.0 ppm (CH_2Cl); ^{29}Si NMR ($CDCl_3$): -70.1 to -66.0 ppm, 11.2 ($OSiMe_3$)

Octakis(3-chloropropyl)octasilsesquioxane [POSS-Cl] was obtained with 12% yield (3.1 g), following the literature procedure [4].

^{1}H NMR ($CDCl_3$): 0.8 ppm ($SiCH_2$), 1.9 ppm (CH_2), 3.5 ppm (CH_2Cl); ^{13}C NMR ($CDCl_3$): 8.1 ppm ($SiCH_2$), 25.1 ppm (CH_2), 45.8 ppm (CH_2Cl); ^{29}Si NMR ($CDCl_3$): -67.0 ppm; Mass Spectrometry: [POSS-T_8-Cl_8 - $C_{24}H_{48}Cl_8O_{12}Si_8$]: m/z 1036.90 ($M^+$).

Synthesis of LPSQ-N_3 and POSS-N_3

LPSQ-Cl (13.7 g), sodium azide NaN_3 (20.56 g) and potassium iodide KI (35.2 g) were placed in a round bottom flask. Freshly distilled DMF (460 mL) was added and the reaction mixture was stirred at room temperature until all substrates were well dissolved. After an hour reaction mixture was heated at 90°C. Reaction progress was monitored using FTIR technique. After two days, C-Cl vibration band disappeared and a new band typical for N_3 vibrations appeared in the FTIR spectrum. The reaction mixture was cooled to room temperature, CH_2Cl_2 was added to the mixture and the solution was washed with water to remove DMF. The solution was dried over magnesium sulfate $MgSO_4$, and filtered, then solvents were removed under reduced pressure. The crude product was dissolved in CH_2Cl_2 and precipitated into hexanes to give yellow, viscous liquid product (12.2 g, 84%, M_n = 6100 g/mol, PDI = 3.0). The crude product was repeatedly precipitated into hexanes until it was possible to divide it into three polymeric fractions (fraction 1 – 0.7 g, fraction 2 – 0.5 g, fraction 3 – 9.3 g) of different molecular weight (Figure S2). Fraction 3 (M_n = 1900 g/mol, PDI = 1.3) was selected for further studies.

^{1}H NMR ($CDCl_3$): 0.05 ppm ($OSiMe_3$), 0.6 ppm ($SiCH_2$), 1.6 ppm (CH_2), 3.2 ppm (CH_2N_3); ^{13}C NMR ($CDCl_3$): 0.3 ppm ($OSiMe_3$), 8.8 ppm ($SiCH_2$), 21.4 ppm (CH_2), 52.1 ppm (CH_2N_3); ^{29}Si NMR ($CDCl_3$): -71.3 to -66.0 ppm, 10.5 ppm ($OSiMe_3$).

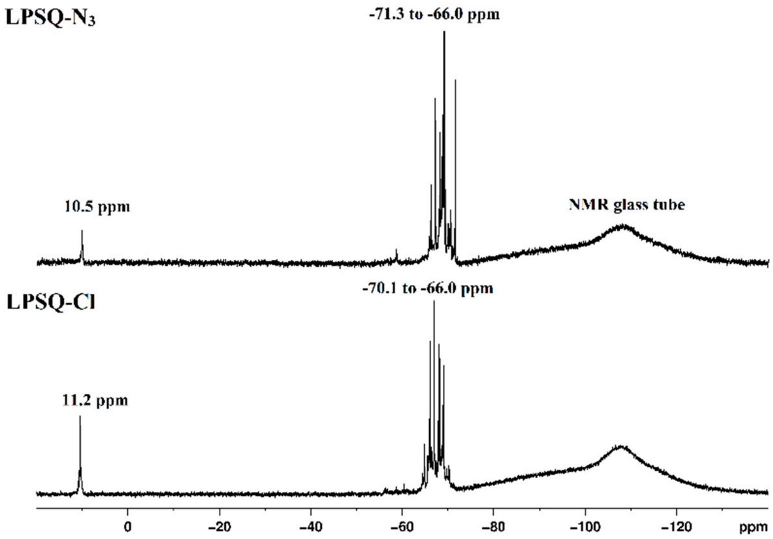

Figure. S1. ^{29}Si NMR ($CDCl_3$) spectra of LPSQ-Cl and LPSQ-N_3 (fraction 3).

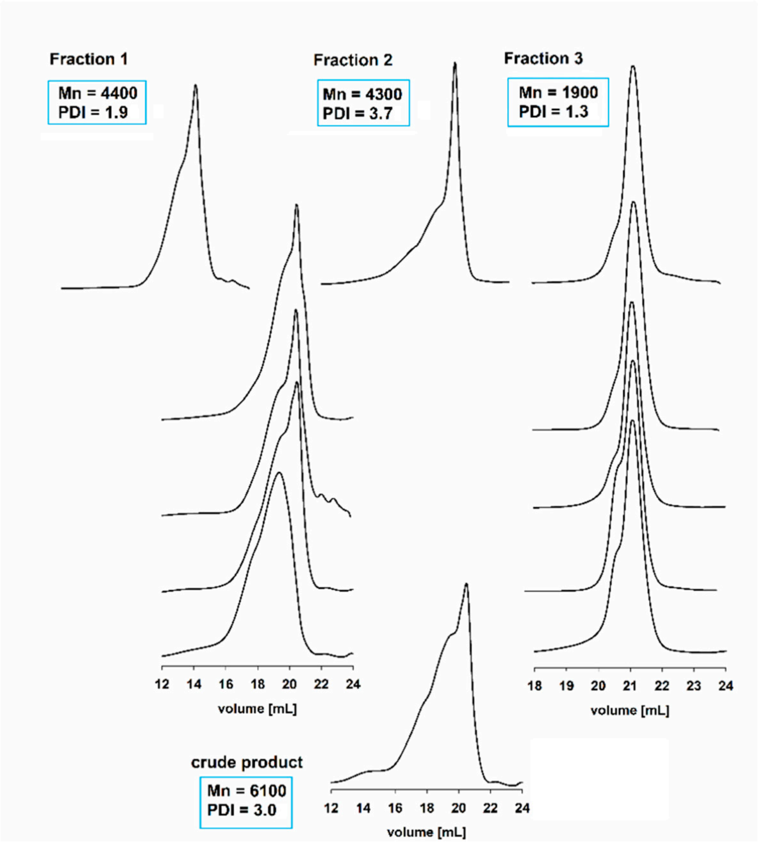

Figure. S2. SEC analysis of LPSQ-N_3: chromatograms of the crude product and three final fractions obtained after precipitation into hexanes (the intermediate fractions are shown for illustration).

Table. S2. Molecular weight estimation based on SEC and NMR (^{1}H, ^{29}Si) analysis of LPSQ-Cl and LPSQ-N_3.

	LPSQ-Cl	n	Crude Product	n	Fraction 1	n	Fraction 2	n	Fraction 3	n
SEC	1400	-	6100	-	4400	-	4300	-	1900	-

PDI	1.3	-	3.0	-	1.9	-	3.7	-	1.3	-
^{1}H NMR	3800	13	6100	22	6700	24	7100	26	5800	21
^{29}Si NMR	3600	13	6800	24	5400	20	4300	15	5900	21

n – degree of polycondensation (the number of repeating units $[Si_2O_3(C_3H_6Cl)_2]$ or $[Si_2O_3(C_3H_6N_3)_2]$).

Degree of polymerization was calculated from the NMR data based on the integration of CH_2Cl (for LPSQ-Cl) or CH_2N_3 (for LPSQ-N$_3$) and $OSiMe_3$ groups (^{1}H NMR) or the integration of $RSiO_{3/2}$ (R=$CH_2CH_2CH_2Cl$ or $CH_2CH_2CH_2N_3$) and $OSiMe_3$ (^{29}Si NMR). The presence of end groups $OSiMe_3$ has been taken into account e.g. for calculated degree of polymerization n=13, estimated molecular weight was M=(13x259)+4x89=3800.

POSS-N$_3$ was obtained in a similar way using POSS-Cl (2 g), NaN_3 (3.2 g), KI (5.2 g) and DMF (200 mL). The product (a viscous, slightly yellow liquid;1 g, Y = 45%) that was isolated from the solution in CH_2Cl_2 by solvent evaporation under reduced pressure, consisted of a mixture of polyhedral silsesquioxanes of different size (POSS-T$_8$-N$_3$ (20.4%), POSS-T$_{10}$-N$_3$ (55.1%) and POSS-T$_{12}$-N$_3$ (24.5%)). Since the size of POSS and the content of prepared product mixture should not affect optical properties of the tested material, further tests were carried out on the above-mentioned mixture of POSS-T$_8$-N$_3$, POSS-T$_{10}$-N$_3$ and POSS-T$_{12}$-N$_3$.

^{1}H NMR ($CDCl_3$): 0.62 ppm ($SiCH_2$), 1.56 ppm (CH_2), 3.17 ppm (CH_2N_3); ^{13}C NMR ($CDCl_3$): 9.3 ppm ($SiCH_2$), 22.3 ppm (CH_2), 52.3 ppm (CH_2N_3); ^{29}Si NMR ($CDCl_3$): -71.6 ppm and-68.8 ppm (POSS-T$_{12}$-$(N_3)_{12}$, with integral ratio 1:2 corresponding to the presence of two different types of silicon atom in the cage of D_{2d} symmetry), -69.2 ppm (POSS-T$_{10}$-N$_3$), -67.1 ppm (POSS-T$_8$-N$_3$), -66.1 ppm (open-cage POSS); Mass Spectrometry: [POSS-T$_8$-N$_3$ - $C_{24}H_{48}N_{24}O_{12}Si_8$]: m/z 1061.20 (M-$N_2^+$); [POSS-T$_{10}$-N$_3$ - $C_{30}H_{60}N_{30}O_{15}Si_{10}$]: m/z 1334.26 (M-$N_2^+$); [POSS-T$_{12}$-N$_3$ - $C_{36}H_{72}N_{36}O_{18}Si_{12}$]: m/z 1606.34 (M-$N_2^+$).

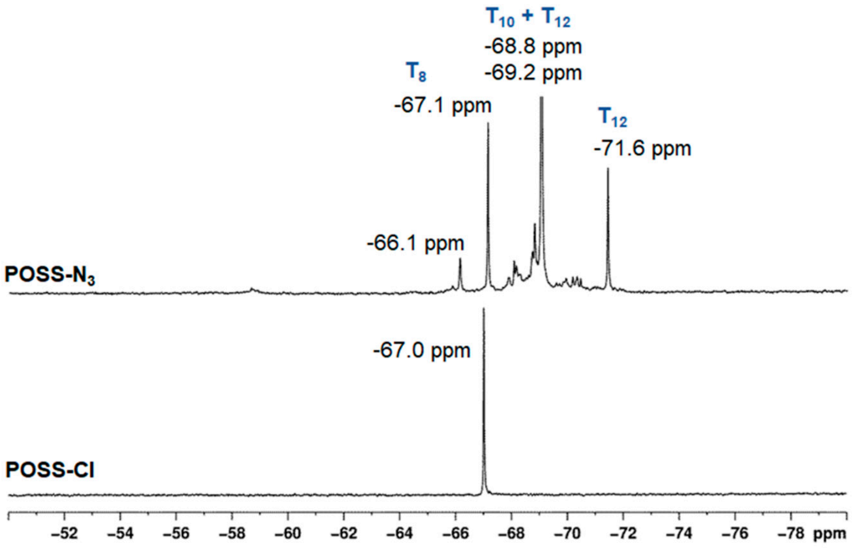

Figure. S3. ^{29}Si NMR ($CDCl_3$) spectra of POSS-Cl and POSS-N$_3$.

Preparation of LPSQ-triazole-Py and POSS-triazole-Py

1-ethynylpyrene (3.33 g) was added to a solution of LPSQ-N$_3$ (2 g) in freshly distilled DMF (400 mL). CuBr (2.12 g) and PMDETA (2.84 mL) was added and the reaction mixture was stirred vigorously at 80°C in an inert gas atmosphere (Ar) to the complete disappearance of N$_3$ vibration band in FTIR spectra. After cooling off to room temperature, CH_2Cl_2 was added and the solution was washed with water to remove DMF. Solvents were removed under reduced pressure and the product was dissolved in CH_2Cl_2 and precipitated into methanol. The obtained yellow precipitate was first purified with silica gel column chromatography, then celite column chromatography (THF used as eluent) and washed with EDTA solution to remove traces of copper. EDS-SEM analysis of the purified material revealed copper concentration below 0.01 %wt (0.01 %atom). The product was then

dissolved in THF and precipitated into cold hexanes. A light yellow solid was obtained after solvents removal (3.68g, 82%).

^{1}H NMR ($CDCl_3$): 0.01 ppm ($OSiMe_3$), 0.8 ppm ($SiCH_2$), 2.2 ppm (CH_2), 4.5 ppm (CH_2-triazole) 7.0 - 7.8 ppm (Ar), 8.4 ppm (NH); ^{13}C HP Dec NMR: -35.2 ppm ($OSiMe_3$), 9.3 ppm ($SiCH_2$), 23.8 ppm (CH_2), 45.1 ppm (CH_2N), 124.5 ppm, 146.8 ppm (Ar); ^{29}Si HP Dec NMR: a broad peak with maximum at -69.1 ppm, 10.5 ppm ($OSiMe_3$).

POSS-triazole-Py was prepared in a similar way using POSS-N_3 (1 g), 1-ethynylpyrene (1.66 g), DMF (200 mL), CuBr (1.06 g) and PMDETA (1.42 mL). The obtained yellow precipitate was purified first with silica gel column chromatography, then celite column chromatography (with CH_2Cl_2 first and then THF used as eluents) and washed with EDTA solution to remove traces of copper. EDS-SEM analysis of purified material revealed copper concentration below 0.01 %wt (0.01 %atom). The product was then dissolved in THF and precipitated into cold hexanes. A light yellow solid was obtained after solvents removal (2.06g, 77%).

^{1}H NMR ($CDCl_3$): 0.8 ppm ($SiCH_2$), 2.2 ppm (CH_2), 4.4 ppm (CH_2-triazole), 7.6 - 8.0 ppm (Ar), 8.2 ppm (NH); ^{13}C NMR ($CDCl_3$): 9.7 ppm ($SiCH_2$), 24.3 ppm (CH_2), 43.5 ppm (CH_2N), 124.5 ppm (Ar), 146.6; ^{29}Si NMR (HP Dec): a broad peak with maximum at -68.5 ppm.

Synthesis of (3-azidopropyl)trimethoxysilane (silane-triazole-Py):

(3-Azidopropyl)trimethoxysilane was prepared according to the modified literature procedure [5]. DMF was used as a solvent and the reaction mixture was heated at 90°C until conversion of Cl groups into N_3 groups was completed (4 days). The reaction mixture was cooled down to room temperature, filtered to remove formed salts (KCl and NaCl). The product was not isolated and the crude solution was used in the next synthetic step. 250 mL of the reaction mixture (the estimated amount of silane-N_3 : 2.5 g) was charged in the Schlenk flask. 1-ethynylpyrene (3.58 g) in DMF (280 ml) was added, followed by the addition of CuBr (3.4 g) and PMDETA (4.6 mL). Reaction mixture was heated at 90°C with vigorous stirring, under inert gas atmosphere for a week. After complete disappearance of N_3 vibration band in FTIR spectra, reaction was cooled down to room temperature. CH_2Cl_2 was added and the solution was washed with water to remove DMF. Solvents were removed under reduced pressure. The crude product was dissolved in CH_2Cl_2 and precipitated into methanol. Yellow precipitate was purified first with silica gel column chromatography, then celite column chromatography (with CH_2Cl_2 first and then THF used as eluents) and washed with EDTA solution to remove traces of copper. EDS-SEM analysis of the purified material revealed copper concentration below 0.02 %wt (0.02 %atom). The product was then dissolved in THF and precipitated into cold hexanes.

^{1}H NMR ($CDCl_3$): 0.05 ppm (CH_3), 0.6 ppm ($SiCH_2$), 2.3 ppm (CH_2), 4.5 ppm (CH_2-triazole) 7.9 - 8.2 ppm (Ar), 8.4 ppm (NH); ^{13}C NMR ($CDCl_3$): -1.8 ppm ($SiCH_3$), 13.7 ($SiCH_2$), 25.5 ppm (CH_2), 53.7 ppm (CH_2N), 122.9-131.3 ppm (Ar), 147.1 ppm (C-NH); ^{29}Si NMR ($CDCl_3$): 2.0 ppm.

2. Characterization:

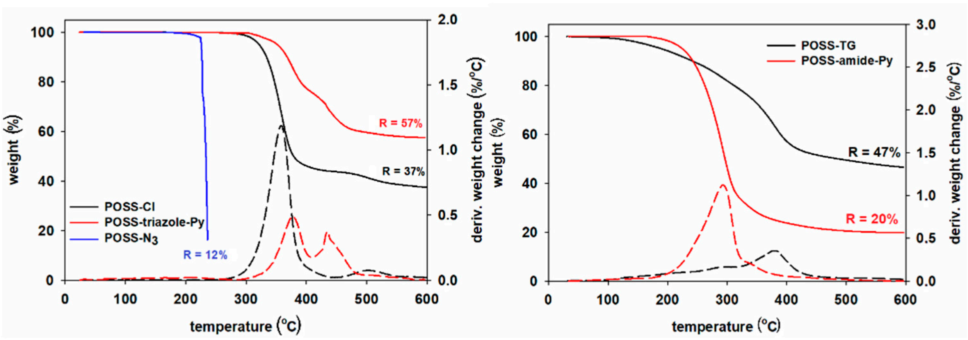

(a) (b)

Figure. S4. Thermogravimetric analysis of a) POSS-triazole-Py and b) POSS-amide-Py (N_2 atmosphere, 10°C/min).

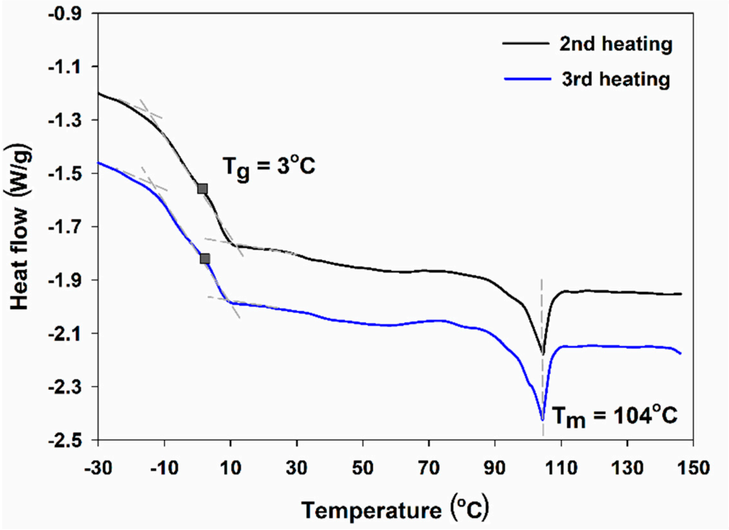

Figure. S5. Thermal analysis of POSS-amide-Py. DSC traces recorded at heating rate 10 °C/min (2nd and 3rd run).

Table. S3. IR band position assignment.

**Vibration Mode**	**LPSQ-TG**	**LPSQ-amide50-Py**	**LPSQ-amide75-Py**	**LPSQ-amide100-Py**
	**Wavenumber [cm^{-1}]**			
ν(C-H)	2600–2400	2900–3050	2950–3050	2900–3000
$\nu_{ring\text{-}asym}$(Si–O)	1116	1140	1136	1151
$\nu_{ring\text{-}sym}$(Si–O)	1040	1040	1036	1039
ω(C-H)aromatic	-	852	854	842
ν OH	3500 - 2700	-	-	-
ν(C=O) in COOH	1707	1736	1740	1745
ν(C=O) in C(O)N	-	1640	1632	1618
ν(C-H) + *bending*(N-H)	-	1514	1521	1521
δ OH in-plane	1423	-	-	-
ν(C–O)	1286	1300	1306	
ν(N– H)	-	3050–3400	3050–3400	3000–3300

**Vibrations**	**LPSQ-Cl**	**LPSQ-N$_3$**	**LPSQ-triazole-Py**
	**Wavenumber [cm^{-1}]**		
ν(C-H)	2900–3000	2900–3000	2900–2950
ν(C–Cl)	700	-	-
ν(N=N=N)	-	2114	-
ν(N=N)	-	-	1434
ν(C=C)	-	-	1550
$\nu_{ring\text{-}asym}$(Si-O)	1124	1124	1112
$\nu_{ring\text{-}sym}$(Si-O)	1036	1044	1044
ω(C-H)$_{aromatic}$	-	-	841

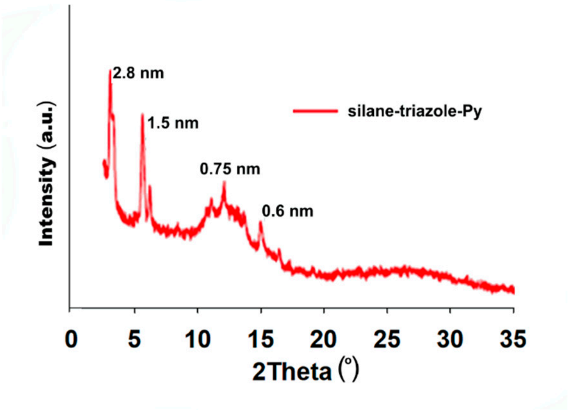

(a)

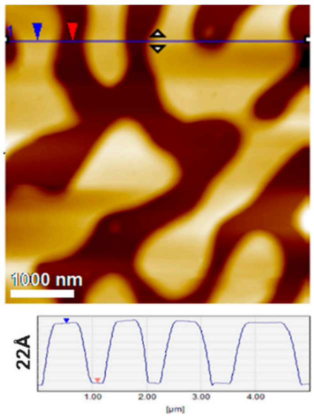

(b)

Figure. S6. (**a**) WAXS diffractogram of silane-triazole-Py, (**b**) AFM height image and cross-sectional analysis of a silane-triazole-Py thin film cast on a silicon support. 22Å.

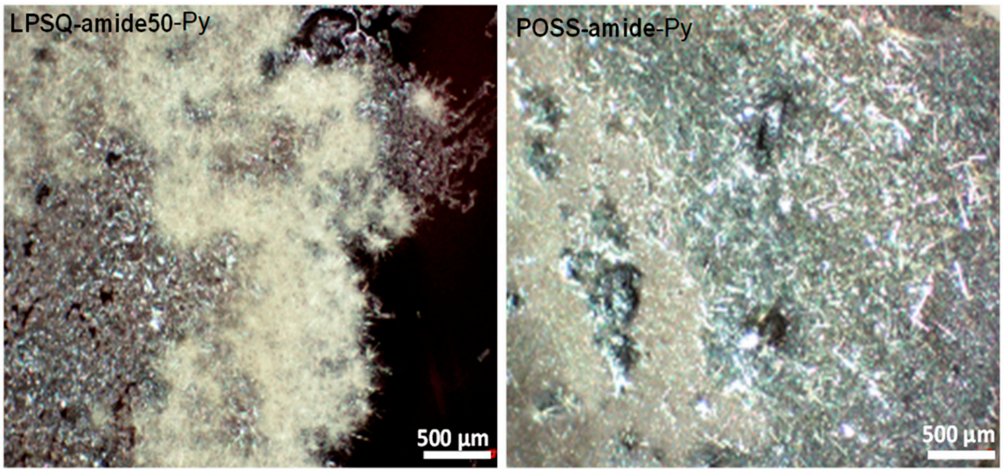

Figure. S7. Reflected light microscopy images of thin films of LPSQ-amide50-Py and POSS-amide-Py.

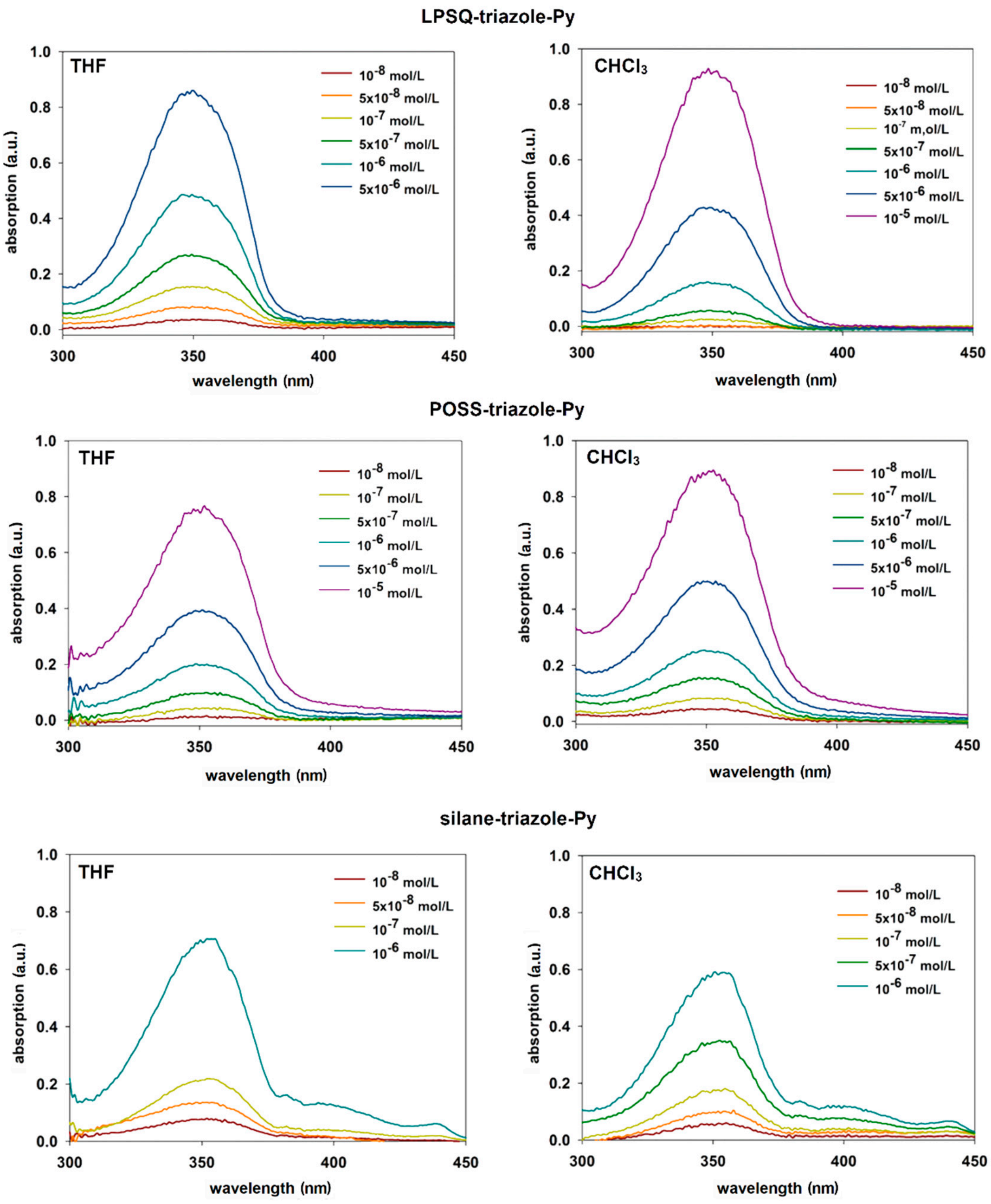

Figure. S8. Absorption spectra of LPSQ, POSS and silane-triazole-Py solutions in THF and chloroform.

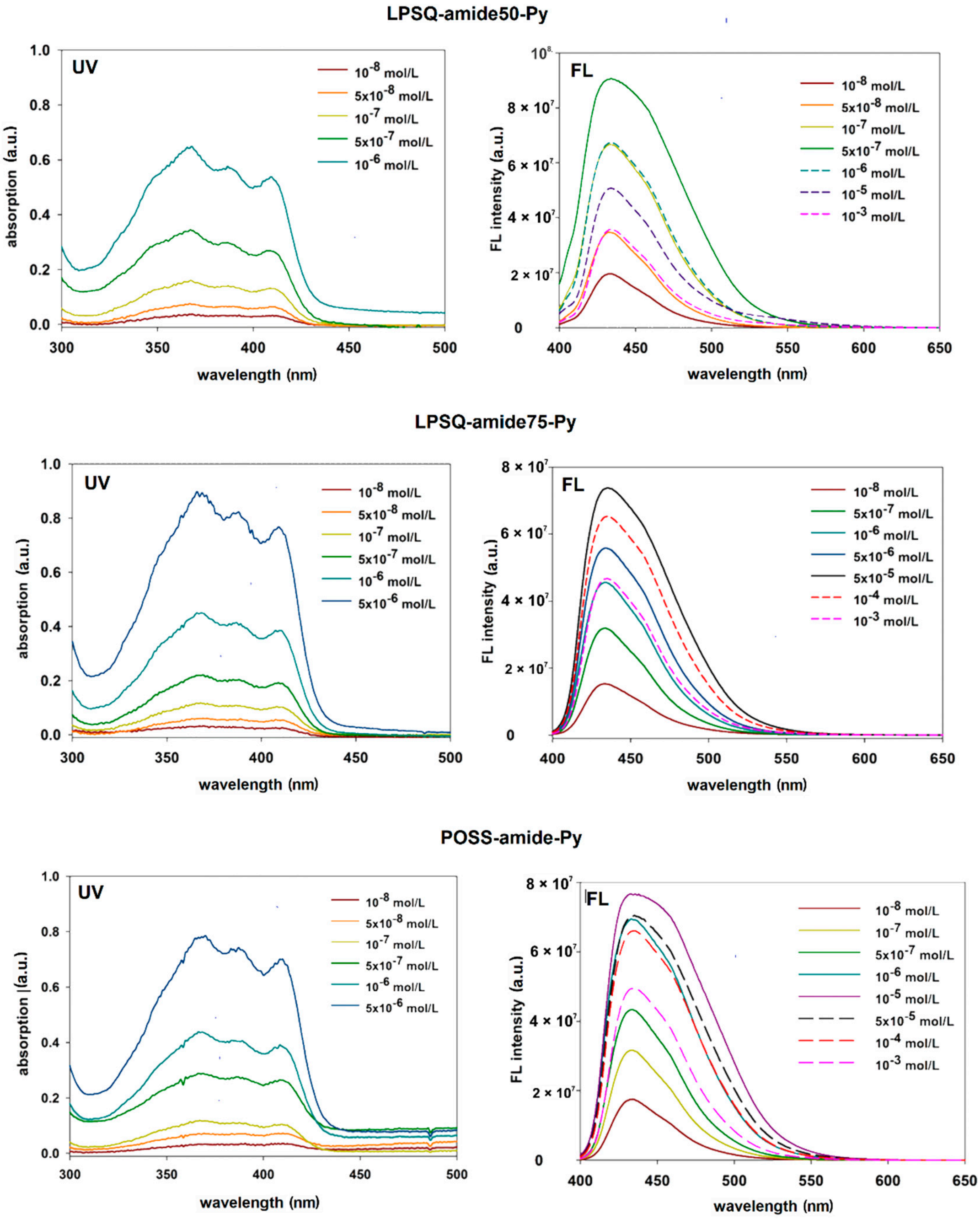

Figure. S9. Absorption and FL emission spectra of LPSQ and POSS-amide-Py in DMF (λ_{ex} = 387 nm).

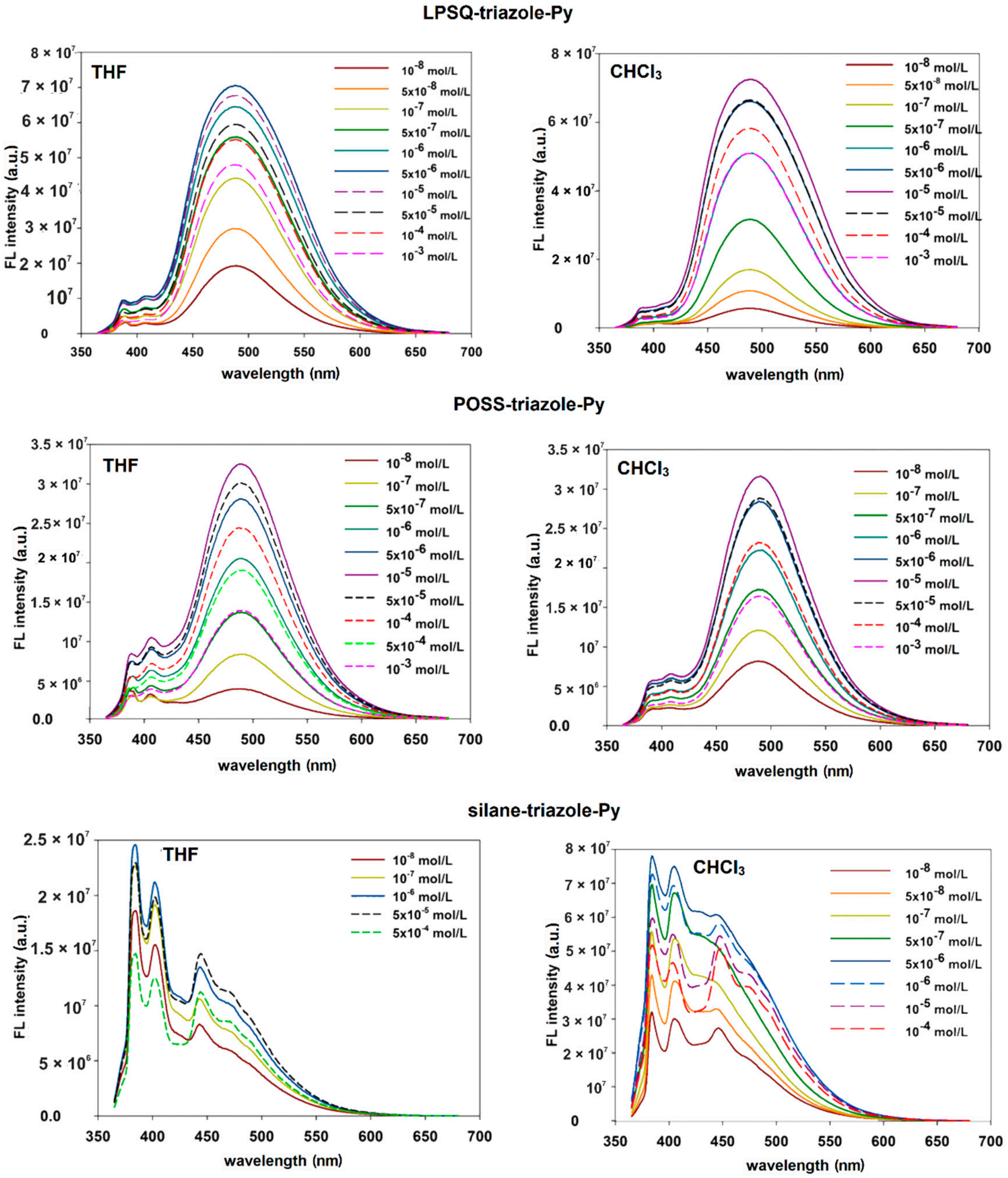

Figure. S10. FL emission spectra of LPSQ, POSS and silane-triazole-Py in THF (350 nm) and $CHCl_3$ (350 nm).

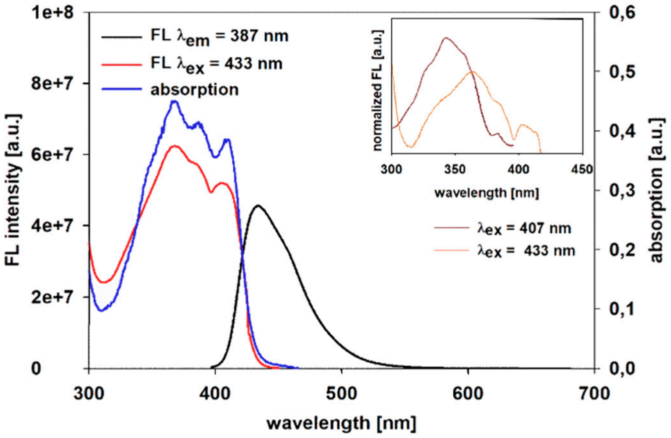

Figure. S11. Emission and excitation fluorescence spectra (inset: absorbance spectra of LPSQ-amide75-Py) recorded for solutions in DMF at c_{Py} =10^{-6} mol/L.

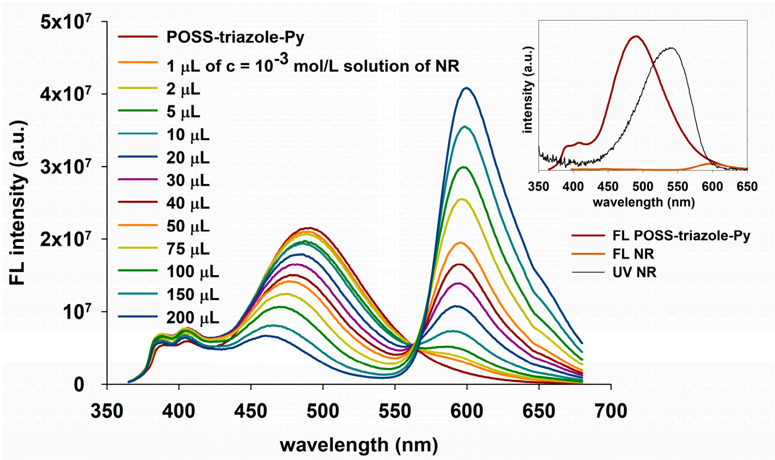

a)

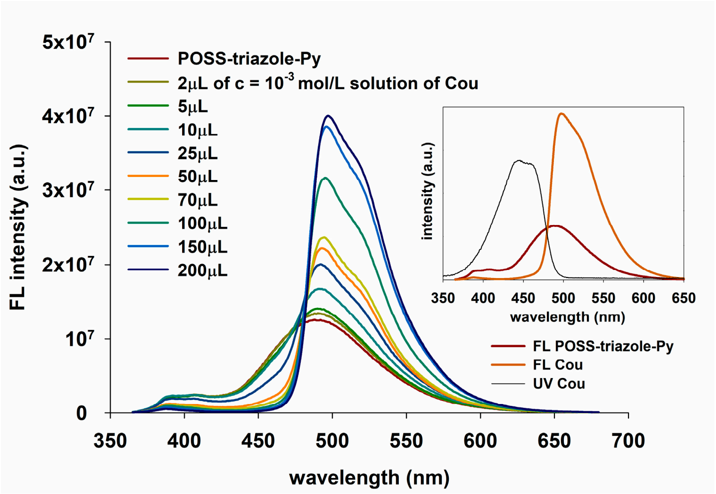

b)

Figure. S12. Energy transfer from POSS-triazole-Py to a) Nile Red and b) Coumarin 6 with dye concentration dependence and spectral overlap of POSS derivatives and dyes. Dye concentration range between $3.3x10^{-7}$ mol/L and $1.6x10^{-5}$ mol/L. Measurements for 3 mL of POSS-triazole-Py solutions at concentration c_{Py} = 10^{-6} mol/L.

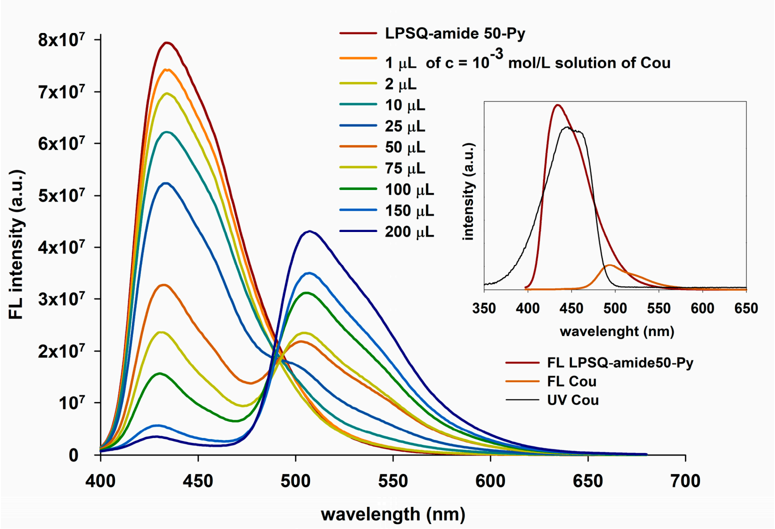

a)

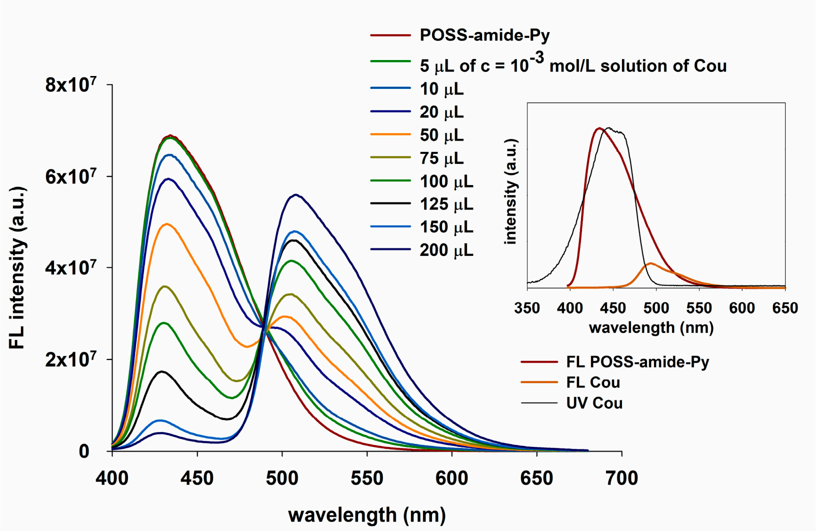

b)

Figure. S13. Energy transfer from LPSQ-amide50-Py and b) POSS-amide-Py to Coumarin 6 with dye concentration dependence and spectral overlap of LPSQ/POSS derivatives and dyes. Dye concentration range between 3.3x10^{-7} mol/L and 1.6x10^{-5} mol/L. Measurements for 3 mL of LPSQ-amide50-Py and POSS-amide-Py solutions at concentration $c_{Py} = 10^{-6}$ mol/L.

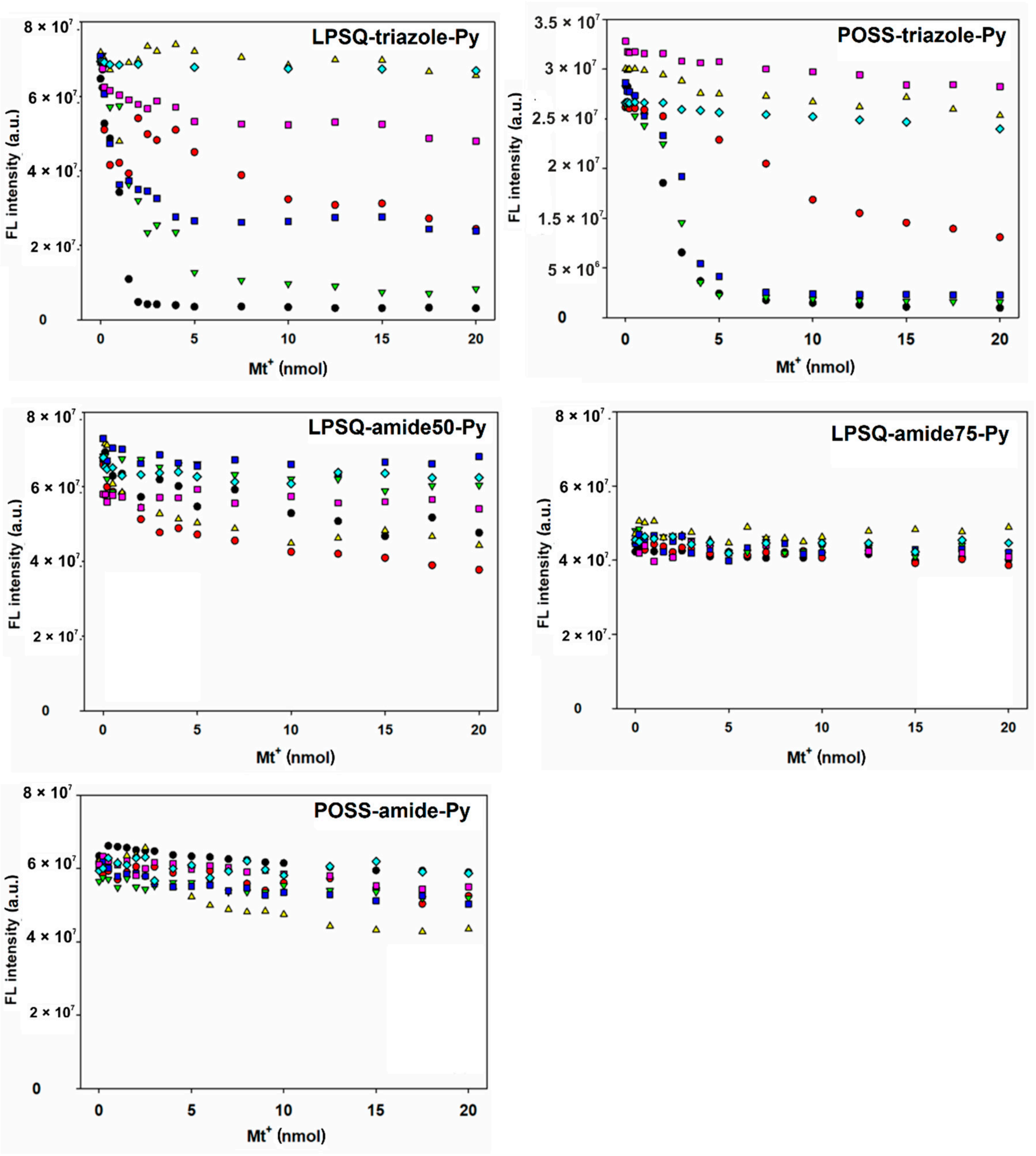

Figure. S14. Changes in FL emission intensity of LPSQ and POSS materials (at c_{Py} =10^{-6} mol/L) containing triazole and amide groups in side chains in the presence in the presence of different perchloric (VII) acid salts: ● – Cu (II), ● – Fe (III), ▼- Ag (I), ▲ – Co (II), ■ – Hg (II), ■ – Zn (II), ♦ - Pb (II)) (solutions at = 10^{-4} mol/L in THF for LPSQ/POSS-triazole-Py or in DMF for LPSQ/POSS-amide-Py).

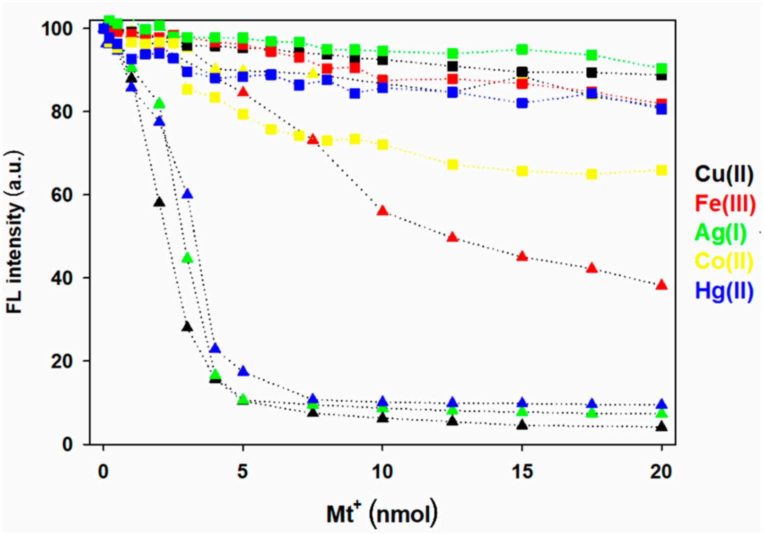

a)

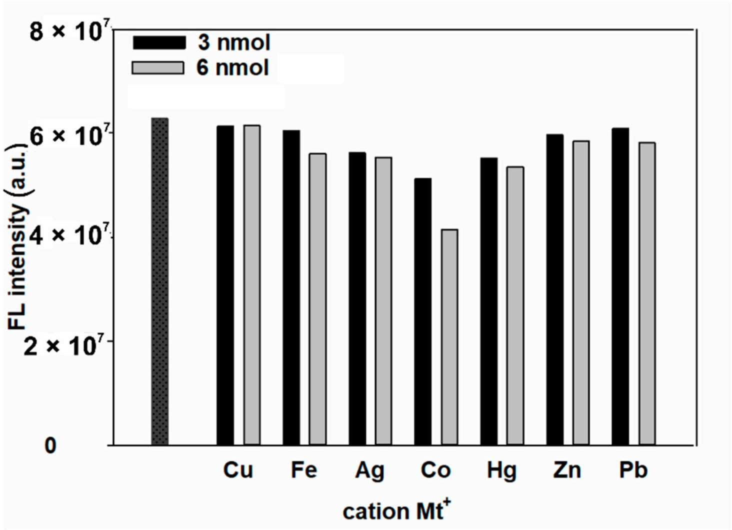

b)

Figure. S15. Comparison of FL emission intensity of a) ▲ - POSS-triazole-Py (in THF) and ■ - POSS-amide-Py (in DMF at c_{Py} = 10^{-6} mol/L) in the presence of increasing concentration of different perchloric (VII) acid salts (solutions at c = 10^{-4} mol/L), b) POSS-amide-Py (3 nmol) in the presence 3 and 6 nmol of different perchloric (VII) acid salts.

Literature:

1. Kowalewska, A.; Nowacka, M.; Tracz, A.; Makowski, T. Supramolecular self-assembly of linear oligosilsesquioxanes on mica – AFM surface imaging and hydrophilicity studies. *Soft Matter* **2015**, *11*, 4818–4829. https://doi.org/10.1039/C5SM00787A.

2. Comerford, J.W.; Clark, J.H.; Macquarrie, D.J.; Breeden, S.W. Clean, reusable and low cost heterogeneous catalyst for amide synthesis. *Chem. Commun*. **2009**, 2562–2564. DOI: 10.1039/b901581g.

3. Krishnan, P.S.G.; He, Ch.; Shang Shang, C.T. Synthesis, characterization, and curing kinetics of novel ladder-like polysilsesquioxanes containing side-chain maleimide groups. *J. Polym. Sci. Pol. Chem.* **2004**, *42*, 4036–4046. https://doi.org/10.1002/pola.20243.

4. Dittmar, U.; Hendan, B.J.; Florke, U.; Marsmann, H.C. Funktionalisierte octa-(propylsilsesquioxane) (3-$XC_3H_6)_8(Si_8O_{12})$ modellverbindungen für oberflächenmodifizierte kieselgele. *J. Organomet. Chem.* **1995**, *489*, 185-194. https://doi.org/10.1016/0022-328X(94)05100-P.

5. Slater, M.D.; Frechet, J.M.J.; Svec, F. In-column preparation of a brush-type chiral stationary phase using click chemistry and a silica monolith. *J. Sep. Sci.* **2009**, *32*, 21–28. doi: 10.1002/jssc.200800493
